# Impact of the V410L *kdr* mutation and co-occurring genotypes at *kdr* sites 1016 and 1534 in the VGSC on the probability of survival of the mosquito *Aedes aegypti* (L.) to Permanone in Harris County, TX, USA

**DOI:** 10.1371/journal.pntd.0011033

**Published:** 2023-01-23

**Authors:** Jonathan R. Hernandez, Shuling Liu, Chris L. Fredregill, Patricia V. Pietrantonio

**Affiliations:** 1 Department of Entomology, Texas A&M University, College Station, Texas, United States of America; 2 Department of Statistics, Texas A&M University, College Station, Texas, United States of America; 3 Harris County Public Health, Mosquito and Vector Control Division (HCPH-MVCD), Houston, Texas, United States of America; Faculty of Science, Ain Shams University (ASU), EGYPT

## Abstract

Harris County, TX, is the third most populous county in the USA and upon detection of arboviruses Harris County Public Health applies insecticides (e.g., pyrethroid-based Permanone 31–66) against adults of *Culex quinquefasciatus* to prevent disease transmission. Populations of *Aedes aegypti*, while not yet a target of public health control, are likely affected by pyrethroid exposure. As this species is a vector of emerging arboviruses, its resistance status to Permanone and the *kdr* mutations in the voltage-gated sodium channel (VGSC) associated with pyrethroid resistance were investigated. We examined females of known genotype at the V1016I and F1534C sites (N = 716) for their genotype at the 410 amino acid position in the VGSC, and for the influence of their *kdr* genotype on survival to Permanone at three different distances from the insecticide source in field tests. Most females (81.8%) had at least one resistant L allele at the 410 position, being the first report of the V410L mutation in *Ae*. *aegypti* for Texas. When only genotypes at the 410 position were analyzed, the LL genotype exhibited higher survivorship than VL or VV. Out of 27 possible tri-locus *kdr* genotypes only 23 were found. Analyses of the probability of survival of tri-locus genotypes and for the V410L genotype using a multivariate logistic regression model including area, distance, and genotype found significant interactions between distance and genotype. When only the most common tri-locus genotypes were analyzed (LL/II/CC, 48.2%; VL/II/CC, 19.1%; and VV/II/CC, 10.1%) genotype had no effect on survival, but significant interactions of distance and genotype were found. This indicated that the V410L *kdr* allele increased survival probability at certain distances. Genotypes did not differ in survivorship at 7.62-m, but LL/II/CC had higher survivorship than VL/II/CC at 15.24- and 22.86-m. The model also identified differences in survivorship among the operational areas investigated.

## Author summary

Preventing *Aedes aegypti* populations from spreading arboviruses in highly populated areas primarily relies upon chemical control with insecticides, such as pyrethroids, applied by public health agencies to target the female mosquito vector. However, the development of insecticide resistance in mosquito populations jeopardizes these vector control strategies. The impact of the different *kdr* mutations conferring resistance to pyrethroids on the survival of *Ae*. *aegypti* in the field is not fully understood. We previously found that two knockdown resistance (*kdr*) mutations in the voltage gated sodium channel (VGSC) did not increase survivorship in field cage tests despite their widespread presence and high frequency. In this paper, we report the first detection of the V410L *kdr* mutation in combination with the V1016I and F1534C genotypes. Most females were detected with *kdr* mutations at one, two or three of these sites in the VGSC. Genotyping females after insecticide application in field cage tests showed that the V410L mutations were associated with higher survivorship only at the 15.24- and 22.86-meter distances from the source of the spray application, as we observed that the LL/II/CC genotype had higher survivorship than the VL/II/CC genotype at these distances.

## Introduction

*Aedes aegypti* (L.) is an efficient primary vector for some of the most medically important arboviruses worldwide, including Zika (ZIKV), dengue (DENV), chikungunya (CHIKV), and yellow fever (YFV) in tropical and subtropical regions of the world [1,2]. The increased distribution of *Ae*. *aegypti* globally exacerbates the risk of arboviral transmission [3,4]. Considered a domestic mosquito, *Ae*. *aegypti* are capable of living in human dwellings, while also preferring human bloodmeals [5], which contributes to increasing arbovirus dissemination [6]. Human arboviral outbreaks caused by *Ae*. *aegypti* have resulted in millions of deaths [7,8]. This species is a competent urban vector for Mayaro virus, an emerging public health concern in South America [9,10], and is also a competent vector for the emerging zoonotic disease Rift Valley fever [11]. Therefore, the need to control of *Ae*. *aegypti* females has led to a reliance on the application of adulticides as one of the major strategies to suppress arboviral disease outbreaks [12]. The most widely synthetic adulticides applied, both by public vector control programs and homeowners are pyrethroids. These act on the central nervous system by modulating the voltage-gated sodium channel (VGSC) preventing channel closure, resulting in insect paralysis and death [13]. Excessive use of pyrethroid space sprays against disease vectors has produced a selection pressure that has caused the development of resistance in mosquito populations worldwide [14–16].

Target site mutations in the VGSC, known as knockdown resistance or *kdr*, represent the most studied mechanism of pyrethroid resistance in insects [17]. The most frequent *kdr* mutations in *Ae*. *aegypti* in the Americas are V1016I and F1534C, which are normally associated with each other [18–21]. A study using a congenic strain (1534C:ROCK) of *Ae*. *aegypti* homozygous resistant only at the F1534C position found that this genotype conferred resistance to both type I and type II pyrethroids [22].

In agreement with this finding, heterologous expression of the F1534C homozygous resistant mutant AaNav1-1 VGSC in *Xenopus* oocytes supports the role of the F1534C mutation in pyrethroid resistance [23]. In contrast, the homozygous resistant V1016I cRNA similarly expressed in the *Xenopus* system did not influence channel sensitivity to type I or II pyrethroids. However, when both resistant genotypes were expressed simultaneously in oocytes (V^1016^I+F^1534^C) the V1016I genotype provided an increased reduction in sensitivity to type I and II pyrethroids compared to the F1534C mutant (F^1534^C) alone [24]. A similar result was found *in vivo*, as heterozygous females at the 1016 and 1534 sites (e.g., VI/FC), resulting from the cross of a double homozygous resistant and a susceptible strain, had intermediate pyrethroid resistance [25].

An additional mutation in the *Ae*. *aegypti* VGSC at the amino acid residue position 410 from valine to leucine (V410L) was reported in 2017 [26], which has since then been reported in combination with the V1016I and F1534C *kdr* genotypes at high frequencies in the Americas [27,28]. However, there are conflicting reports on the influence of the V410L mutation in *Ae*. *aegypti* resistance to pyrethroids. *In vitro* electrophysiological assays support that the V410L *kdr* genotype alone reduces sensitivity to type I and type II pyrethroids [26]. The reduced sensitivity to permethrin was further enhanced by the presence of the F1534C resistant genotype, suggesting an additive effect of the two mutations on permethrin resistance [26]. In contrast, female mosquitoes of the congenic LKR strain (a resistant strain from La Mesa backcrossed into the pyrethroid susceptible Rockefeller strain) that was triple homozygous resistant for the V410L, V1016I, and F1534C *kdr* genotypes did not confer higher levels of resistance to seven insecticides compared to the resistance levels of 1534C:ROCK, a congenic strain with only the F1534C resistant genotype [22,29]. Resistance to these insecticides (cyhalothrin, cypermethrin, etofenprox, DDT, permethrin, cis-permethrin, and trans-permethrin) ranged from 7- to 16- fold when compared to the susceptible Rockefeller strain [22,29]. Harris County, TX, which includes the City of Houston, is the third-most populous county in the United States with over 4.73 million residents reported in 2020 [30]. The county’s humid, subtropical climate lies within the ideal habitat range of *Ae*. *aegypti* [1]. Houston is one of the most vulnerable areas for flooding events, especially the densely populated downtown area [31]. Concerns about flooding in Harris County have increased after natural disasters such as Hurricane Harvey in 2017. Flooding in the Harris County area could increase the number of mosquito oviposition sites, which would increase the area at risk of exposure to mosquito-borne diseases [32, 33]. This rapidly growing metropolitan area thus requires the Harris County Public Health, Mosquito and Vector Control Division (HCPH-MVCD) to provide public health services to protect country residents from arbovirus outbreaks. HCPH-MVCD conducts yearlong surveillance and testing for several arboviruses transmitted by mosquitoes, including West Nile and St. Louis encephalitis viruses as well as DENV, CHIKV, and ZIKV [34].

Upon detection of arboviruses in one of their Mosquito and Vector Control Operational Areas (MVCOA), HCPH-MVCD schedules ground-based ultra-low volume (ULV) applications to reduce vector mosquito populations. These treatments have primarily been against *Culex quinquefasciatus* upon detection of West Nile virus [35]. Adult females of *Cx*. *quinquefasciatus* are controlled by alternating ULV applications of the pyrethroid Permanone 31–66 (31% permethrin; 66% piperonyl butoxide (a synergist cytochrome P450 enzymes inhibitor)) and the organophosphate malathion utilizing HCPH-MVCD trucks mounted with London Fog 18–20 ULV sprayers that are able to reach effective spray distances up to 30.48 m [35]. While HCPH-MVCD has not yet targeted either *Ae*. *aegypti* or *Aedes albopictus* for control with synthetic pesticides, these species would be targeted if diseases such as those caused by ZIKV, DENV, or CHIKV viruses were detected within Harris County. If targeted application against *Aedes* mosquitoes were necessary, chemical interventions are planned to be performed with Permanone 31–66 using ULV handheld sprayers, each capable of reaching areas that trucks cannot, such as backyards. In addition, piperonyl butoxide is the only metabolic enzyme inhibitor found in formulated products with the active ingredient permethrin available for public health use. ULV handheld foggers used by HCPH-MVCD can reach distances up to 15.24 m and can be utilized indoors and outdoors [36]. Although *Ae*. *aegypti* mosquitoes have not been targeted for control, the frequent pyrethroid applications against *Culex quinquefasciatus* could have contributed to resistance development as both species are present in Harris County and these *Ae*. *aegypti* populations carry *kdr* mutations [21,37].

Previously, we analyzed in *Aedes aegypti* populations of Harris County the frequency of the two most reported *kdr* mutations in the Americas that had been associated with permethrin resistance, V1016I and F1534C [21]. These *kdr* mutations were widely distributed across Harris County, with 77% of all female mosquitoes analyzed carrying the double homozygous resistant genotype II/CC. While the field-collected mosquitoes exhibited increased survivorship (at three distances and up to 22.86 m) compared to the susceptible Orlando-strain females, there were no differences in survivorship among the different genotypes detected at the 1016 and 1534 residue sites. These results indicate that there may be other *kdr* genotypes that could explain the survivorship of the females in the field tests. The resistant V410L genotype in combination with V1016I and F1534C *kdr* genotypes has been detected in the United States, with the triple resistant genotype (LL/II/CC) being associated with permethrin resistance [27,38].

This present study builds upon earlier work by investigating the presence of the V410L mutation in Harris County and determining if it contributes to in-field resistance when occurring in mosquitoes with known V1016I and F1534C genotypes. Herein we report the first detection of the resistant V410L mutation for Texas and the increased survivorship of homozygous tri-locus genotypes.

## Materials and methods

### Mosquitoes

The *Ae*. *aegypti* Orlando insecticide susceptible strain from Florida, U.S., originated in 1952 and since then has been maintained as a laboratory colony, which is used as a standard for baseline toxicological testing in this species [39, 40]. At the HCPH-MVCD in Harris County this strain has been maintained since 2015.

For egg field collection and rearing details, refer to Hernandez et al., 2021 [21]. Egg collection was conducted between November 2017 and August 2019, and the areas of origin of the mosquitoes and test dates were: Area 23, November 9, 2017; Area 419, September 9, 2018; Area 53, October 11, 2018; Area 73, November 6, 2018; Areas 45 and 75, July 16, 2019; Areas 601 and 806, August 6, 2019.

For field bioassays *Ae*. *aegypti* females were two-to-five-day-old either F_0_ generation obtained from eggs collected from the field, or from the laboratory Orlando-strain colony [21].

Genomic DNA used in this study was prepared from 716 females of *Ae*. *aegypti* that had been genotyped for the V1016I and F1534C sites in a previous study from our laboratory [21].

Briefly, DNA was isolated from individual mosquitoes using the ZYMO Quick-DNA Microprep kit, following a modified version of the manufacturer’s protocol with a one-hour incubation period in the DNA lysis buffer that was used to obtain greater DNA yields. The concentration and quality of each DNA sample were determined using an automatic microplate reader (Tecan Infinite M200). Genomic DNA was diluted to 10 ng/μL for genomic DNA analyses.

### Field cage tests

Field cage tests that simulated real mosquito control scenarios were used to detect susceptibility of *Ae*. *aegypti* to Permanone 31–66 at three distances were described in Hernandez et al. [21]. Briefly, the field test plot utilized a 3 x 3 design with 3 rows of three-cage-carrying posts positioned at 7.62-, 15.24-, and 22.86-m downwind of the insecticide application source. Within each distance, posts were positioned at 7.62-m from each other as subsamples. Each post had at least three cages, with at least two cages containing female mosquitoes from the field and one cage with pyrethroid-susceptible Orlando-strain females. Three similar posts with cages were placed upwind from the treatment source as negative controls (expected to survive), and to correct the mortality in the treated cages, if needed [41]. All tests used the maximum application rate for permethrin of 0.008 kg a.i./ha. Each single pass test had an operator applying Permanone 31–66 with a Colt-4 ULV Handheld Fogger Sprayer (London Fog, Minneapolis, MN). The direction of the pesticide treatment was perpendicular to the walking path [21].

### Genomic DNA analyses

To detect the *kdr* genotypes in the VGSC at the 410 position (V410L) in females from the field tests, we used a DNA melting curve analysis of allele-specific PCR products carried out in 96-well plates using a QuantStudio 6 Pro system (Applied Biosystems, Foster City, CA) and diagnostic primers for the V410L mutations, as described by Saavedra et al. [20]. Peaks were analyzed using the Thermo Fisher’s Design and Analysis v 2.3 software (Thermo Fisher Scientific Inc, Waltham, MA, US). For the V410L mutation detection, the 10 μL reaction mixture contained 5 μL of PowerUP SYBR Green Master Mix (Thermo Fisher Scientific, MA), 0.2 μM of the V410fw and 410rev primers (Table 1), 0.17 μM of the L410fw (Table 1), and 1 μL (10 ng) of gDNA template. PCR conditions consisted of a 3 min 95°C activation step, followed by 35 cycles at 95°C for 10 s, 60°C for 10 s, and 72°C for 30 s, with a final extension step of 15 s at 95°C. The melting curves were determined from 65°C to 95°C, with an increase of 0.2°C every 1 s. Based on the melting curves, a single peak at 80°C corresponded to the 113 bp product of the homozygous resistant mutant (LL), a peak at 83°C corresponded to the 133 bp product of the homozygous susceptible wild-type (VV), and two peaks, one at each of both temperatures corresponded to a heterozygous mutant (VL). Each PCR plate included i) a positive control to detect the wild-type susceptible genotype (VV) using the gDNA of one Orlando strain female and primers V410fw and 410rev, ii) one negative control that used the same primers and nuclease-free water instead of genomic DNA. All samples were analyzed in triplicate.

**Table 1:** Sequences of primers used for detection of the *kdr* V410L in the *Aedes aegypti* VGSC. Primers feature base pair mismatches introduced at the third base from 3’ end to increase allele specificity (italics); the diagnostic differential nucleotide is in bold, underlined [42].

*Kdr* Site	Primer Name	Primer Sequence (5’ → 3’)
V410L	V410fw	GCGGGCAGGGCGGCGGGGGCGGGGCCATCTTCTTGGGTTCGTTCTACC*G*T**G**
	L410fw	GCGGGCATCTTCTTGGGTTCGTTCTACC*A*T**T**
	410rev	TTCTTCCTCGGCGGCCT*C*TT

### Statistical analyses

Statistical analyses were performed using R software (R version 4.0.2, R Foundation for Statistical Computing, Vienna, Austria) and GraphPad Prism (San Diego, CA). Frequency and the survival rate of female mosquitoes were calculated for all subgroups of each predictor including genotype, distance, area, and post position. A Pearson Chi-Square test was used to test the univariate association between the survivorship and each individual predictor. The Fisher exact test was used for pairwise comparisons. Multivariate logistic regression models were fitted to assess the relationship between survival rate and all the predictors simultaneously. First, due to the sparsity of data, including low counts of certain genotypes and/or at specific distances, we used the Least Absolute Shrinkage and Selection Operator (LASSO) logistic regression model to select the most correlated predictors for survival rate [43]. The LASSO technique is widely used with sparse data since it can improve the quality of predictions by shrinking regression coefficients compared to models estimated with regular unpenalized maximum likelihood approaches which may tend to overfit the data. Some coefficients are set to zero based on LASSO, and therefore parsimony is also achieved for the selected model. The final model included genotype, distance, area and the interaction between genotype and distance. Second, since the confidence interval estimates using the LASSO method can be quite complicated, one common practice is to do statistical inference based on the selected model. Hence, we refitted the logistic model by excluding subgroups of the combinations of genotypes and distances with less than or equal to 16 females (the sample size of 16 corresponds to a margin of error of 0.25) or those subgroups with LASSO coefficients equal to zero. Next, we evaluated the goodness-of-fit using pseudo-R square and area under the curve (AUC) to evaluate our final model performance. Lastly, sensitivity analysis was performed by fitting the model using full data or using other thresholds of low count genotypes. We compared the model fitting results including parameter coefficients and goodness-of-fit to validate our final model. A multiple comparison adjustment was applied to control for type I errors when comparing more than two groups. The odds ratio of each predictor was calculated and visualized using a forest plot. P-values less than 0.05 were considered statistically significant.

The impact of the factors, area, genotype, and distance on the probability of survival of the mosquitoes was assessed through a logistic regression model. The model of the log-odds, where *p* equals probability of survival, is:

$$log\left(\frac{p}{1-p}\right)=\beta_{0}+\;\beta_{1}*Genotype\;+\;\beta_{2}*Distance\;+\;\beta_{3}*Area+\;\beta_{4}*Genotype*Distance$$

## Results

### Genotype analysis

**Fig 1:**
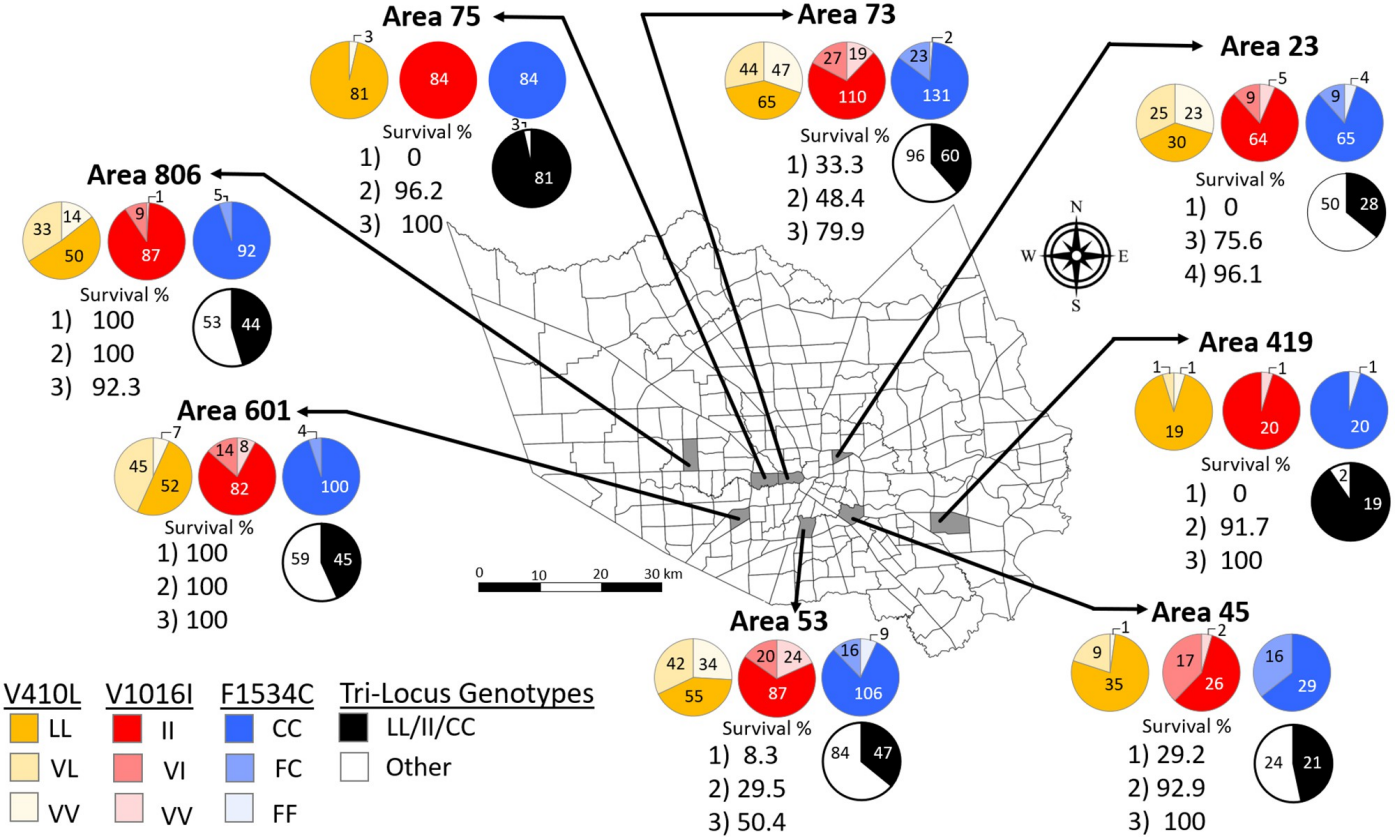
Proportion of *kdr* mutations in female mosquitoes shown by operational control area in Harris County. The pie charts represent the genotypic frequencies for each of the *kdr* mutations detected in individual females of *Ae*. *aegypti* collected for the eight operational areas in Harris County, adapted from Hernandez et al. [21]. Proportions of the genotypes at the 410 site are in shades of yellow (LL, VL, VV), and those for the 1016 (II, VI, VV) and 1534 (CC, FC, FF) sites previously genotyped are represented by shades of red and blue, respectively. In each pie chart, the lightest color intensity represents the wild-type, susceptible genotypes (410 VV; 1016 VV; or 1534 FF) and the darkest color indicates the resistant genotype (410 LL; 1016 II; 1534 CC), with the intermediate color intensities corresponding to the heterozygotes (410 VL; 1016 VI; 1534 FC). For each area, the proportion of tri-locus homozygous resistant (LL/II/CC) females is shown in black, while the proportion of all other tri-locus genotypes at the 410, 1016, and 1534 genotypes are in white. The number of genotypes per area is indicated on the corresponding color on each pie chart. Survivorship data (Survival %) for each area (shown in columns below pie charts) by distance from the spray application [1) 7.62, 2) 15.24, 3) 22.86, and 4) 38.1 m] was previously reported [21] but is shown here for clarity. Map source: edited in QGIS v.3.22.1 software (www.qgis.org). The layers for county (Map service: Harris County boundary masked) (https://www.arcgis.com/home/item.html?id=a8aa2ef4067348c79ccea62857a2f623) and for Harris County operational area boundaries (MVCDOperational_Areas) (https://www.arcgis.com/home/item.html?id=87a4455991c146259cf6c2e2384e8b71) were created by Harris County Public Health and are publicly available. There are no special restrictions or limitations on the terms of use of the layers applied to this map.

To determine the influence of a third *kdr* mutation in the VGSC on the survivorship of females tested in the Harris County’s field cage tests, 716 *Ae*. *aegypti* females previously individually analyzed for the V1016I and F1534C *kdr* mutations were genotyped for the 410 position, and the results are shown in Fig 1 and Table 2. This V410L mutation was widespread across all eight areas analyzed and present at high frequency, with 54.1% of the females (*N* = 387) possessing the homozygous resistant LL genotype. Across the eight areas, the presence of the LL genotype ranged from 38.5% in area 23 to 96.4% in area 75 (Fig 1 and Table 2). When compared to the homozygous resistant genotypes at the V1016I and F1534C sites (II and CC), the resistant LL genotype at the 410 position was less frequent in seven of the eight operational areas, except area 45. Area 45 was unusual in that females had a higher frequency of the 410 LL genotype (77.8%) than either the resistant 1016 II (57.8%) or 1534 CC (64.4%) genotypes (Fig 1 and Table 2).

**Table 2:** Tri-locus VGSC genotypes at sites 410, 1016, and 1534 detected in *Ae*. *aegypti* females of Harris County by operational area.

	Mosquito Control Operational Areas								Total
	23	45	53	73	75	419	601	806	
**Genotypes**	n; (%)	n; (%)	n; (%)	n; (%)	n; (%)	n; (%)	n; (%)	n; (%)	n; (%)
VV/VV/FF	1; (1.3)	0; (0)	4; (3.1)	2; (1.3)	0; (0)	1;(4.8)	0;(0)	0;(0)	8; (1.1)
VV/VV/FC	1;(1.3)	0;(0)	4;(3.1)	5; (3.2)	0;(0)	0;(0)	0;(0)	0;(0)	10; (1.4)
VV/VV/CC	2; (2.6)	0;(0)	6; (4.6)	8; (5.1)	0;(0)	0;(0)	4; (3.8)	0;(0)	20; (2.8)
VV/VI/FC	2; (2.6)	0;(0)	4; (3.1)	7; (4.5)	0;(0)	0;(0)	0;(0)	0;(0)	13; (1.8)
VV/VI/CC	0;(0)	0;(0)	2; (1.5)	2; (1.3)	0;(0)	0;(0)	2; (1.9)	0;(0)	6; (0.8)
VV/II/FC	0;(0)	0;(0)	0;(0)	0;(0)	0;(0)	0;(0)	0;(0)	1; (1.0)	1; (0.1)
VV/II/CC	17; (21.8)	1; (2.2)	14; (10.7)	23; (14.7)	3; (3.6)	0; (0)	1; (1.0)	13; (13.4)	72; (10.1)
VL/VV/FF	0; (0)	0; (0)	4; (3.1)	0; (0)	0; (0)	0; (0)	0; (0)	0; (0)	4; (0.6)
VL/VV/FC	0; (0)	0; (0)	1; (0.8)	0; (0)	0; (0)	0; (0)	0; (0)	0; (0)	1; (0.1)
VL/VV/CC	1; (1.3)	0; (0)	2; (1.5)	0; (0)	0; (0)	0; (0)	1; (1.0)	0; (0)	4; (0.6)
VL/VI/FC	5; (6.4)	5; (11.1)	4; (3.1)	9; (5.8)	0; (0)	0; (0)	3; (2.9)	1; (1.0)	27; (3.8)
VL/VI/CC	2; (2.6)	0; (0)	5; (3.8)	8; (5.1)	0; (0)	0; (0)	5; (4.8)	4; (4.1)	24; (3.4)
VL/II/FF	1; (1.3)	0; (0)	0; (0)	0; (0)	0; (0)	0; (0)	0; (0)	0; (0)	1; (0.1)
VL/II/FC	1; (1.3)	0; (0)	0; (0)	0; (0)	0; (0)	0; (0)	0; (0)	0; (0)	1; (0.1)
VL/II/CC	15; (19.2)	4; (8.9)	26; (19.8)	27; (17.3)	0; (0)	1; (4.8)	36; (34.6)	28; (28.9)	137;(19.1)
LL/VV/FF	0; (0)	0; (0)	1; (0.8)	0; (0)	0; (0)	0; (0)	0; (0)	0; (0)	1; (0.1)
LL/VV/FC	0; (0)	0; (0)	0; (0)	1; (0.6)	0; (0)	0; (0)	0; (0)	0; (0)	1; (0.1)
LL/VV/CC	0; (0)	2; (4.4)	2; (1.5)	3; (1.9)	0; (0)	0; (0)	3; (2.9)	1; (1.0)	11; (1.5)
LL/VI/FC	0; (0)	11;(24.4)	3; (2.3)	1; (0.6)	0; (0)	0; (0)	1; (1.0)	2; (2.1)	18; (2.5)
LL/VI/CC	0; (0)	1; (2.2)	2; (1.5)	0; (0)	0; (0)	0; (0)	3; (2.9)	2; (2.1)	8; (1.1)
LL/II/FF	2; (2.6)	0; (0)	0; (0)	0; (0)	0; (0)	0; (0)	0; (0)	0; (0)	2; (0.3)
LL/II/FC	0; (0)	0; (0)	0; (0)	0; (0)	0; (0)	0; (0)	0; (0)	1; (1.0)	1; (0.1)
LL/II/CC	28; (35.9)	21; (46.7)	47; (35.9)	60; (38.5)	81; (96.4)	19; (90.5)	45; (43.3)	44; (45.4)	345;(48.2)
**Total**	78	45	131	156	84	21	104	97	716

**Fig 2:**
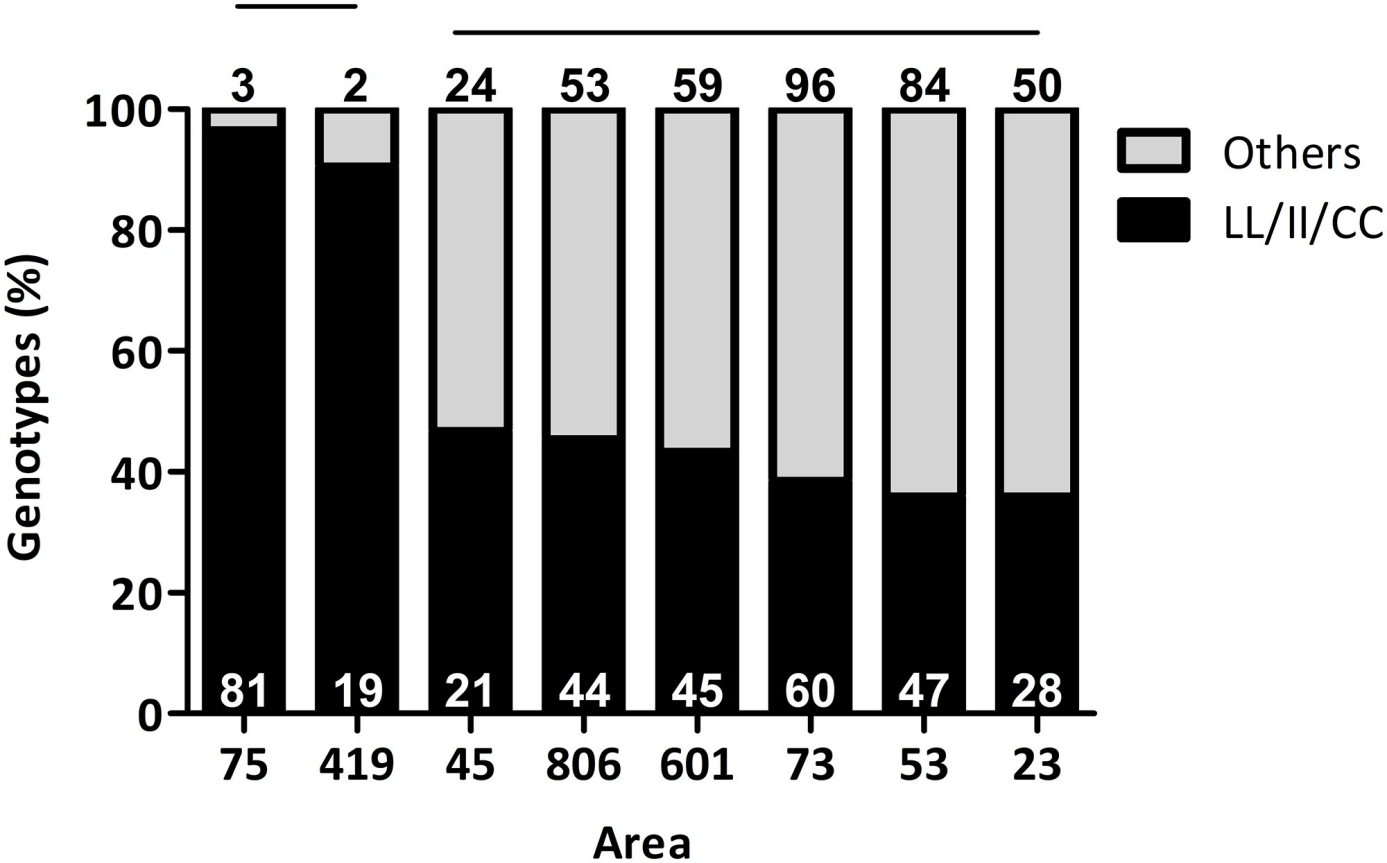
Percentage of tri-locus homozygous resistant genotypes at the 410, 1016 and 1534 sites (LL/II/CC) vs other tri-locus genotypes detected per area. Percentage of the tri-locus (LL/II/CC) *kdr* mutation in females of *Ae*. *aegypti*. Significant differences were detected among the proportion of LL/II/CC genotypes observed across all areas (Chi-square; *P* < 0.0001). Horizontal lines above bars indicate areas in which the percentage of genotypes are not significantly different as per pairwise comparison analyses (Fisher Exact test; *P* < 0.05). White numbers in the black bars represent the number of females of the LL/II/CC genotype and black numbers above the bars represent all other genotypes observed in each area.

The tri-locus genotypes found were not equally distributed in all areas (Table 2 and Figs 1 and 2). Among the different operational areas, area 53 had the highest variety of genotypes observed, with seventeen genotypes detected within that area, followed by areas 23 and 73 with thirteen genotypes each, and area 601 with eleven different genotypes. Areas 45, 75, and 419 had the lowest variety of genotypes, with seven, two, and three, respectively (Table 2). The triple-*kdr* genotype (LL/II/CC) was the most abundant genotype observed in all areas, ranging from 35.9% in areas 23 and 53, to 96.42% in area 75 (Fig 1 and Table 2). This genotype comprised 48.2% of all females tested and the proportions of LL/II/CC genotypes among the eight different areas were statistically different (Fig 2; Chi-square test, *P*-value < 0.0001). This genotype was followed in frequency by VL/II/CC (19.1%), VV/II/CC (10.1%), VL/VI/FC (3.8%), and VL/VI/CC (3.4%) (Table 2). Variation was also observed for some of these most abundant genotypes, for example, VL/II/CC ranged from 0 in area 75 to 34.6% in area 601 and VV/II/CC ranged from 0 in area 419 to 21.8% in area 73. Eleven other tri-locus genotypes (VV/VV/FF, VV/VV/FC, VV/VV/CC, VV/VI/FC, VV/VI/CC, VL/VV/FF, VL/VV/CC, LL/VV/CC, LL/VI/FC, LL/VI/CC, and LL/II/FF) ranged between 0.3% and 2.8% of all females tested. The remaining seven tri-locus genotypes, VV/II/FC, VL/VV/FC, VL/II/FF, VL/II/FC, LL/VV/FF, LL/VV/FC, and LL/II/FC each had only one female representative. These were detected in areas 23, 53, 73, and 806, likely due to high variety of observed genotypes within these areas and indicates the sampling of egg collections was representative of the population.

Analyses of female mosquitoes for the V410L genotypes only supported that areas differ in the number of homozygous resistant (LL), homozygous susceptible (VV) or heterozygous resistant genotypes (VL) (S1 Fig; Chi square, *P* < 0.0001). The statistical analyses with Fisher Exact test revealed two groups of areas with similar proportions for the VV, VL and LL genotypes, as follows: areas 806, 601, 53, 73, and 23 (S1 Fig) had more VV and VL individuals compared to areas 75 and 419. Area 45 was intermediate between the two groups.

Females were then compared for the tri-locus homozygous *kdr* genotype (LL/II/CC) versus to all other tri-locus genotypes. All areas had more LL/II/CC individuals than any of the other genotypes detected (Fig 2 and Table 2; Chi square, *P* < 0.0001). Pairwise comparisons among the different areas for their similarity in the proportion of LL/II/CC genotypes revealed two groups, as follows: 1) areas 75 and 419, and 2) areas 45, 806, 601, 53, 73, and 23 (Fig 2). Group 1 had an overall higher proportion of LL/II/CC females compared to the other areas, with 90.5% and 96.4% of the females detected being the resistant genotype in areas 419 and 75, respectively. The proportion of LL/II/CC females from group 2 ranged from 35.9% in areas 23 and 53, to 46.7% in area 45; the area 45 was intermediate being not significantly different from the two groups.

### Linkage disequilibrium

Linkage disequilibrium (LD) analyses between pairs of alleles at the three *kdr* loci coding for V410L and V1016I, V410L and F1534C, and V1016I and F1534C, respectively, were conducted for each area (S1–S3 Tables). Area 75 could not be analyzed for linkage disequilibrium due to reaching fixation for the resistant genotypes II at 1016 and CC at the 1534 site. When analyzing linkage disequilibrium between the V410L and V1016I sites, six areas, areas 23, 419, 53, 73, 601 and 806, had significant LD with Χ^2^ *P* values < 0.05 and D’ values ranging from 0.422–0.999 (S1 Table). This supports that these areas are in LD for the 410 and 1016 loci except area 45, which had a higher proportion of V410L *kdr* alleles compared to the V1016I *kdr* alleles (Fig 1).

LD analyses for the V410L and F1534C sites, found that only three areas, 419, 53, and 73, had significant LD with Χ^2^ *P* values < 0.05 and D’ values ranging from 0.493–0.999 (S2 Table), which is likely due to the increased distance between these loci which would support more crossover events. Finally, the LD analyses for the V1016I and F1534C sites, found that areas 23, 419, 53, 73, 45, 75, 601 and 801, had significant LD with Χ^2^ *P* values < 0.001 and D’ values ranging from 0.507–0.999 (S3 Table). The analyses indicate that when comparing alleles at the different loci pairs, V1016I and F1534C loci were in strong, but not perfect, linkage disequilibrium.

### Impact of V410L genotype on survivorship

**Fig 3:**
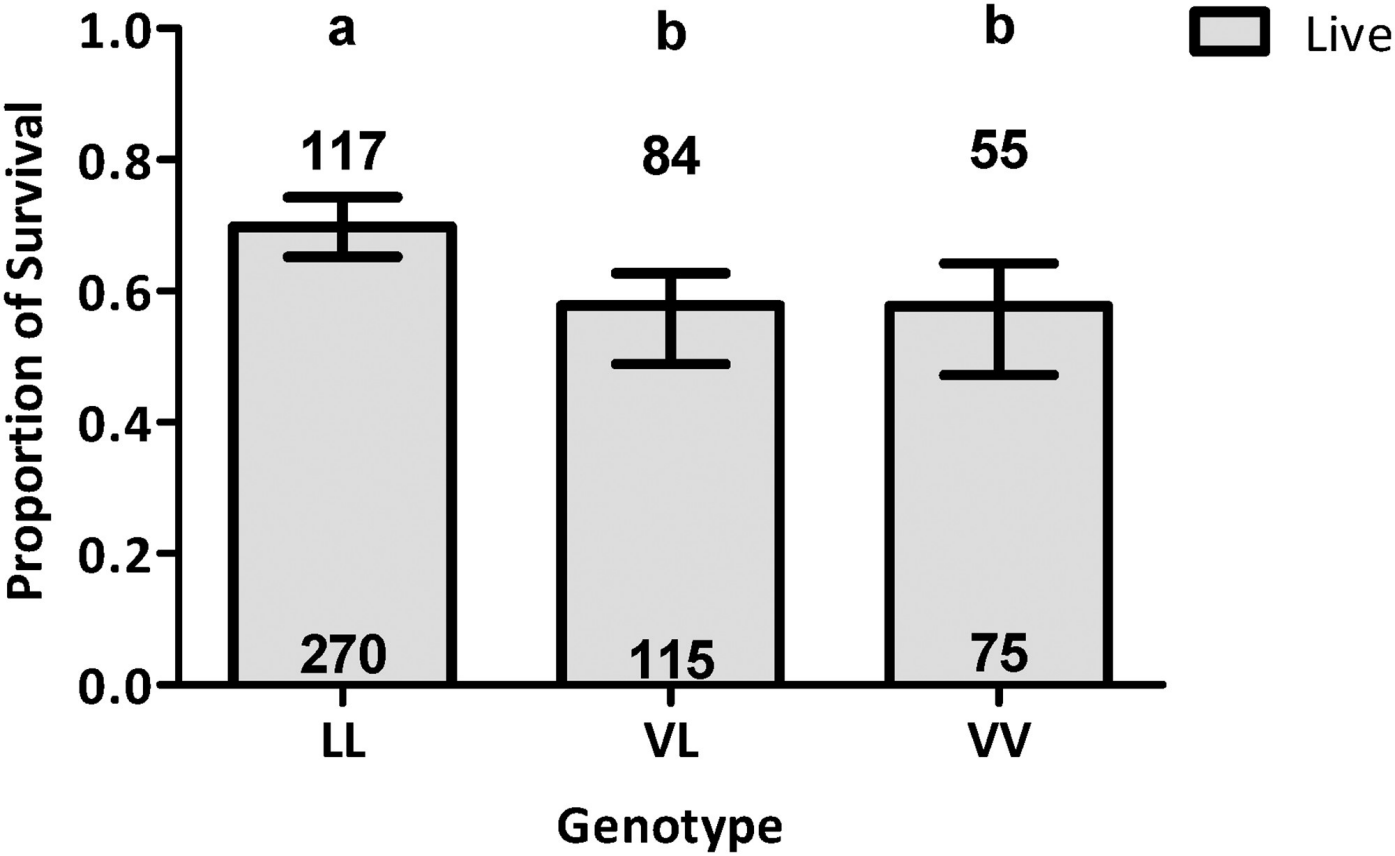
Impact of genotype at the 410 site on female survival after Permanone 31–66 tests. Estimated proportion of surviving females of *Ae*. *aegypti* by genotypes at the 410 site. There were highly significant differences in the proportion of surviving females among VV, VL and LL genotypes (*P* = 0.0037). The histogram shows the estimated proportions of surviving females ± CI, and different letters (a-b) above bars indicate differences in the proportions of surviving females (Fisher’s Exact Test). Numbers in the grey bars of the histogram are the surviving females, and the numbers above the histogram represent the number of dead females.

We then asked if the presence of the V410L genotypes had an overall impact on survivorship regardless of their area of origin or distance from the treatment source (Fig 3).

After adjusting for differences in area and distance, there was a significant difference in the survivorship of mosquitoes among the three genotypes detected (Fisher’s exact test, *P* = 0.004; Fig 3). The survival proportion for LL was significantly higher than the survival proportions for VL and VV (Fisher’s Exact Test, *P* = 0.044 and 0.0134, respectively). The survival proportions for VL and VV were not significantly different (Fisher’s Exact Test, *P* = 0.074; Fig 3).

We then attempted to estimate the effect of genotype on survivorship at every distance. Survivorship of each tri-locus genotype at every distance tested also could not be statistically evaluated, due to the lack of representative genotypes at different distances from the sprayer in all areas studied (S4 Table). We thus analyzed for the V410L site (independently of genotypes at 1016 and 1534) the genotype effect on survivorship for each distance, but for all areas combined (S2 Fig). At 7.62 m, there were no differences in survivorship among genotypes (S2A Fig; Chi-square, *P* = 0.0516) from the Permanone 31–66 application. Genotypes at the 410 position showed differences in survivorship at 15.24 m (S2B Fig, Chi-square, *P* = 0.0001) and at 22.86 m (S2C Fig, Chi-square, *P* = 0.0001) with the homozygous resistant, LL, having higher survivorship. At 38.1 m, the test did not detect significant differences in survivorship for the three genotypes (S2D Fig; Chi-square, *P* = 0.8878).

### Effect of the most common tri-locus kdr genotypes on survivorship by distance

**Fig 4:**
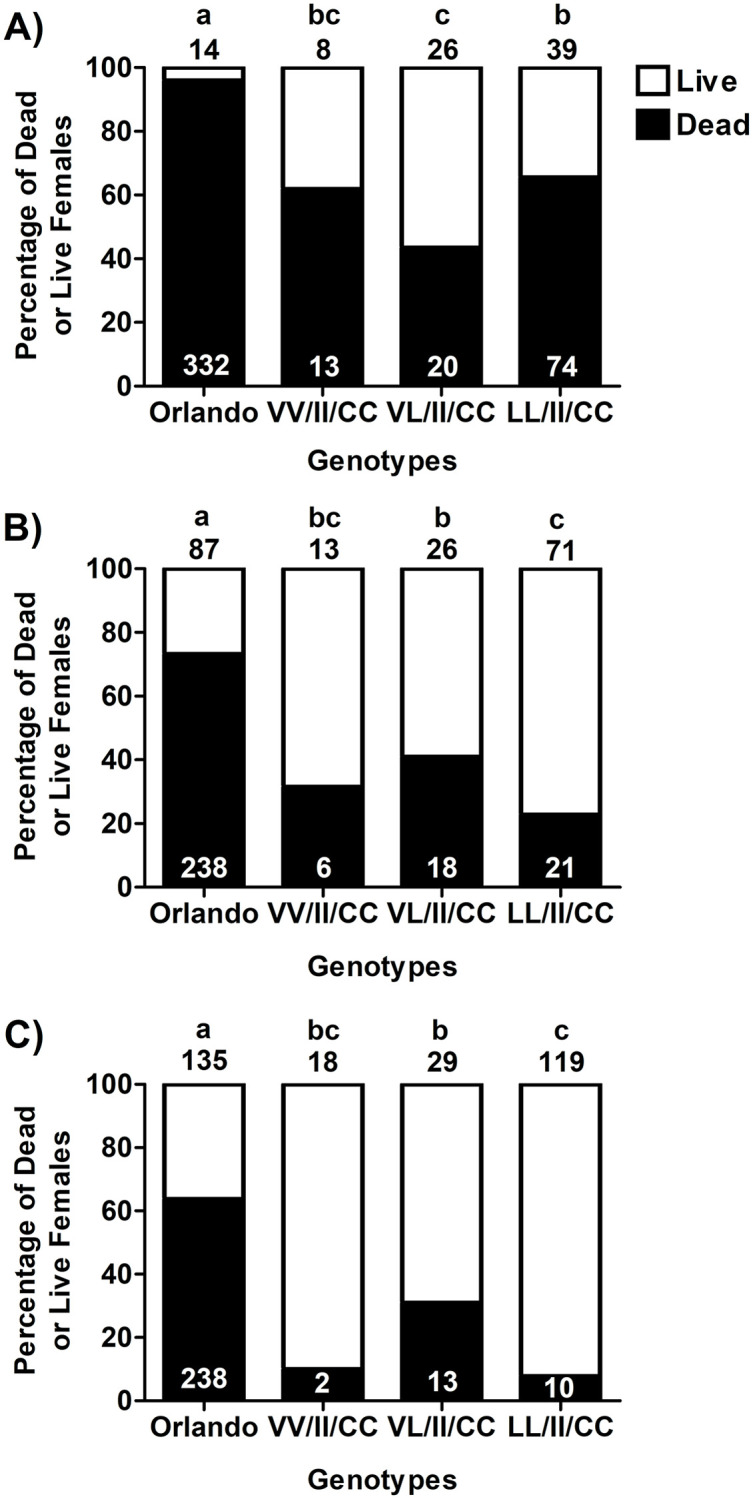
Influence of the most common tri-locus genotypes (410, 1016 and 1534) on the survivorship of females at each distance after Permanone 31–66 tests. Panels show results of each of the tested distances from the Permanone 31–66 application source, as follows: (A) 7.62 m, (B) 15.24 m, and (C) 22.86 m. Orlando strain susceptible females are used as positive controls. Numbers in black above each bar represent the genotyped mosquitoes that survived, and numbers in white within the black zones are the genotyped mosquitoes that died. Different letters (a-c) above bars indicate differences in the proportions of surviving females (Fisher’s Exact Test).

We then analyzed the survivorship of the three most common genotypes (VV/II/CC, VL/II/CC, LL/II/CC) compared to the susceptible Orlando strain controls on the same cage post at each distance. The choice for those three genotypes was due to the lack of representations of other genotypes when considering separation by different distances (S4 Table). The Orlando females had the highest mortality at each distance compared to the field collected females (Fig 4). At all distances the VV/II/CC genotype showed no significant difference to the VL/II/CC and LL/II/CC genotypes, while the latter two genotypes were significantly different from each other at each distance (Fig 4A–4C). The low number of the VV/II/CC (N = 60) females present may explain their similar survivorship to the VL/II/CC (N = 132) and LL/II/CC (N = 334) genotypes, as their low number were further stratified by distance. At the 7.62 m distance from the Permanone 31–66 source, survivorship of the field collected females ranged from 34.5% (LL/II/CC) to 56.5% (VL/II/CC) while the Orlando females only had 5% survival (Fig 4A), the lowest of all distances. The VL/II/CC genotype had significantly higher survivorship compared to the LL/II/CC genotype (Fisher’s exact test, *P* = 0.0129; Fig 4A). However, the survivorship of the triple-resistant LL/II/CC was higher compared to the VL/II/CC genotype at 15.24 m (Fig 4B; Fisher’s exact test, *P* = 0.0292) and 22.86 m distances (Fig 4C; Fisher’s exact test, *P* < 0.0001) from the spray application. This analysis indicates that there are differences among the genotypes when compared at the different distances and the differences in mortality with respect to the Orlando strain were more pronounced at the furthest distance (Fig 4C).

### Analysis of post position effect on survivorship

To determine if the differences in survivorship from the field cage tests may be attributed post position, the Orlando and field collected females with the three most common genotypes were analyzed by post number (S3 Fig). Post position refers to the placement of the posts within each distance, where post I is the closest to the beginning of the spraying and post III is the one closest to the ending of the spraying. At each post position, the Orlando females had the lowest survivorship compared to the field collected females. However, only at post position I, the VL/II/CC and triple homozygous resistant genotype (LL/II/CC) had higher survivorship, being significantly different from the Orlando females, while the VV/II/CC genotype was intermediate (S3A Fig). At post positions II and III, the three field collected genotypes, VV/II/CC, VL/II/CC, and LL/II/CC had significantly higher survivorship proportions compared the Orlando females (S3B and S3C Fig).

The most frequent genotypes VV/II/CC, VL/II/CC, and LL/II/CC were analyzed for survivorship differences among the different areas (S4 Fig). When areas 601 and 806 were not considered in the analyses due to their high survivorship, there were no significant differences in survivorship among areas for the LL/II/CC and VV/II/CC genotypes (S4A and S4C Fig), but females with the VL/II/CC genotype from area 53 (S4B Fig) had significantly lower survivorship compared to most areas. Area 53 also had the highest number of different genotypes detected in this study (S4 Fig and Table 2). Area 419 only had one female of genotype VL/II/CC and area 45 had 4 such females; statistically both areas, 419 and 45, were intermediate in survivorship between area 53 and areas 23 and 73; the latter two did not differ (S4 Fig). The results from this analysis support that areas influence survivorship even when females from different areas are of the same genotype, in this case VL/II/CC.

### Impact of genotype on survivorship using logistic regression modeling

Logistic regression models for the 410 genotype alone and for the tri-locus genotypes (S4 Table) were fit to the data with interaction terms to determine the influence of genotypes on the survival of female mosquitoes. When evaluating the influence of the 410 genotype on survivorship with all data considered, genotype had a significant positive effect on survival (LR Chi-square, *P* = 0.002) (S4 Table, Panel A, top). The increase in survival of the 410 site genotype remained significant even when excluding "distance of 38.1 m" (only for area 23) (S4 Table, Panel A, middle; LR Chi-square, *P* = 0.0024) and number of females per genotype that were less than 16 (S4 Table Panel A bottom; LR Chi-square, *P* = 0.0024). The increased “distance” from the sprayer was the most significant factor enhancing survivorship (S4 Table, Panel A, top; LR Chi-square, *P* < 0.0001), regardless of the exclusion of data at 38.1 m distance (S4 Table, Panel A, middle; LR Chi-square, *P* < 0.0001) and of genotypes represented by low counts (16 or less females) (S4 Table, Panel A, bottom; LR Chi-square, *P* < 0.0001). The significant interaction between the 410 site genotype and distance regardless of the consideration of these mentioned exclusions, indicated that the effect of genotype on survivorship will be influenced by distance from the sprayer (S4 Table; LR Chi-square, *P* < 0.0001).

We tried to measure the effect of genotypes at the 410 site on survival in the context of the genotypes at the other two *kdr* sites, 1016 and 1534 (S4 Table). For this we analyzed the 23 genotypes resulting from the three *kdr* sites (tri-locus genotypes) for 716 females (which include females of area 23 from one test conducted at the distance of 38.1 m instead of 15.24 m). We found there was a significant difference in the survival proportions among the 23 genotypes (Fisher’s Exact Test, *P* = 0.0020; S4 Table). However, the low numbers of certain genotypes detected, such as LL/VV/FF or LL/II/FC each represented by one female (Table 2), prevented to estimate the differences in survivorship between specific tri-locus genotypes (pairwise comparisons could not be performed).

When testing the tri-loci genotypes, distance remained the most significant indicator of survivorship, regardless of excluding data at 38.1 m (S4 Table, Panel B; LR Chi-square, *P* < 0.0001) and genotypes with less than 16 samples. When all samples and 23 different genotypes were considered, there were significant differences in survivorship (S4 Table, Panel B, top; LR Chi-square, *P* < 0.0020). When the distance of 38.1 m was removed, we observed that the triple loci genotypes still exhibited differences in survivorship (S4 Table, Panel B, middle; LR Chi-Square, *P* < 0.0007). However, when excluding the genotypes with less than 16 females, we observed that the 3 most frequent remaining genotypes (LL/II/CC, VL/II/CC, and VV/II/CC) did not appear to have an influence on survivorship (S4 Table, Panel B, bottom; LR Chi-square, *P* = 0.4240).

The interaction between the tri-locus genotypes and distance when considering all females tested supported that the 23 different genotypes at different distances experienced differences in survivorship (S4 Table, Panel B, top; LR Chi-square, *P* = 0.0025). When removing the 38.1 m distance, the interaction between genotype and distance remained significant (S4 Table, Panel B, middle; LR Chi-square, *P* = 0.0009). Upon excluding genotypes and distance interactions with less than 16 samples, the interaction between genotype and distance remained significant (S4 Table, Panel B, bottom; LR Chi-square, *P* = 0.0002).

**Fig 5:**
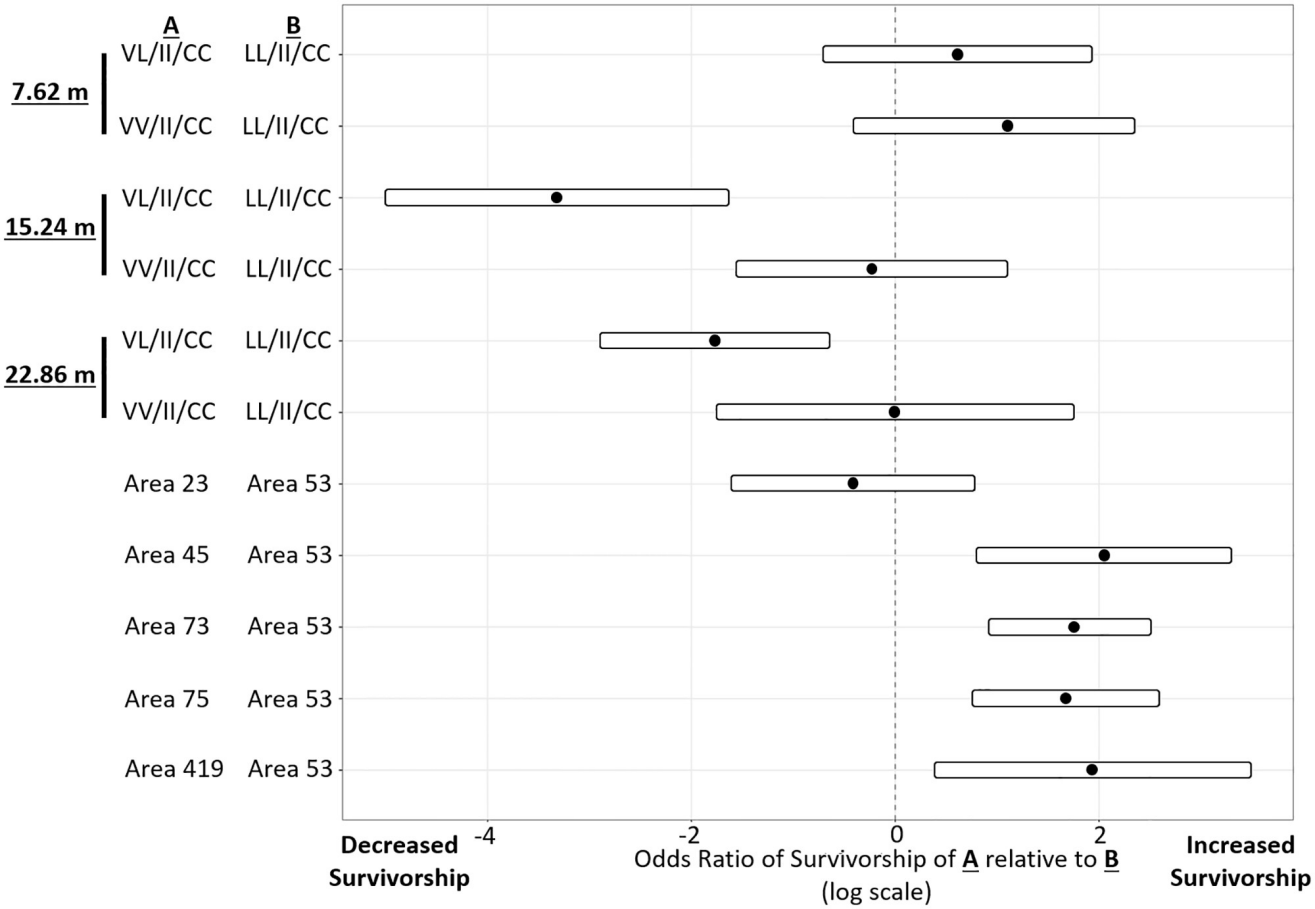
Forest plot of the final model showing odds ratio for female survivorship by the interaction of distance and the tri-locus genotypes, and by operational area. Genotypes or areas under **A** are compared to the genotype or area reference level, indicated by **B**; the genotype with the highest survivorship, LL/II/CC, and Area 53, with the lowest survivorship, were chosen as the reference levels for this plot.

Based on multivariate logistic regression, models with the triple mutation genotype (LL/II/CC as the reference level genotype with higher survivorship), distance (7.62 m as the reference level), area (area 53 as the reference level for higher mortality) and the interaction between “genotype and distance” were significantly associated with survivorship of mosquitoes. Distance again was the most predictive factor for survivorship in the field cage tests, followed by the interaction between distance and genotype (S5 Table). The effects of interaction between genotype and distance, and area effect are visualized using a forest plot (Fig 5). The multiple comparisons supported that genotype of females had little impact on the survival rate at the 7.62 m distance (Fig 5 and S5 Table). At the 15.24- and 22.86-meter distances, females with the triple homozygous resistant mutant LL/II/CC genotype, had a significantly higher survival rate compared to those with VL/II/CC (Fig 5 and S5 Table). When differences by area were considered in the model, we observed that areas 45, 73, 75, and 419 had increased survivorship compared to the reference area 53 but not area 23 (Fig 5 and S5 Table). All pairs of different distances are significantly different in survival rate regardless of genotype (S6 Table). That is, survival rate will increase dramatically when distance gets further.

### Model evaluation

We evaluated the goodness-of-fit of our final model. The McFadden pseudo-R square is 0.54 and area under the curve (AUC) is 93.9%, indicating excellent goodness-of-fit. The LASSO (Least Absolute Shrinkage and Selection Operator) logistic regression model showed that the survivorship predictors with largest coefficients are distance, occurrences of genotype VL/II/CC at distances of 15.24 or 22.86 m, and area, and therefore validates our final model fitting results. We also performed a sensitivity analysis by fitting the same model using the full data and by fitting the model without “area”. The findings from the full data model are consistent with our final model with similar significant terms and similar goodness-of-fit. However, due to the sparsity, the standard errors for many subgroups become extremely large, making the estimate unstable. Therefore, our final model is more reliable compared to full data analysis. A model excluding area has similar model estimates for genotype and distance as well as their interactions, but the goodness-of-fit is poorer with a much smaller pseudo-R square (0.22) and AUC (79.2%), which means our model outperforms this area-excluding model. In short, although areas 601 and 806 have almost 100% survival rates, adding data of these areas to our final model does not change our conclusions but rather significantly improves the analysis provided by the model.

## Discussion

The goal of this study was to investigate the effect of the *kdr* V410L mutation, the combined genotypes at 410, 1016 and 1534 sites in the VGSC, and other test variables on mosquito survival after Permanone application. First, we found high frequencies of the V410L *kdr* mutation associated with pyrethroid resistance, manifested as higher than expected survivorship at 7.62 m in most operational areas investigated (Figs 1 and 2). In similar trials with susceptible *Aedes aegypti* 95–100% mortality is expected at 6 m [44].

This is the first detection of the V410L *kdr* mutation in Texas, which was found frequently in combination with the V1016I and F1534C genotypes (Table 2 and Fig 1). In our previous publication we found that while there were high frequencies of the two latter *kdr* alleles, a logistic regression model found no significant association between genotype and survivorship [21], which motivated the current study.

### Genotype variation among areas of Harris County

Indeed, 81.8% of all females genotyped had at least one resistant allele at the 410 position (Table 2 and Figs 1 and 2). These results match previous studies which had found high frequencies of the V410L genotype in combination with V1016I and F1534C genotypes throughout the world, including the Americas and Africa [20,26–28,45–47].

Of all genotypes detected, the most frequent across all areas in Harris County was the triple homozygous resistant (LL/II/CC) which comprised 48.1% of all mosquitoes tested (Table 2 and S4 Fig). The most common *kdr* alleles in the Americas are 410L + 1016I + 1534C [27]. The presence of the homozygous resistant LL genotype at the 410 site likely contributed to the high female survivorship at the 15.24- and 22.86-m distances from the spray application source (Figs 3 and 4 and S2 and S5 Tables, bottom panel and S6 Table, 2^nd^ and 3^rd^ panels).

The distribution of V410L genotypes was not even across all areas (Figs 1 and 2). Among the areas analyzed, areas 75, 419, and 45 had the highest proportion of LL females at the 410 site (S1 Fig). For most areas, the proportion of *kdr* genotypes followed a pattern in which the F1534C resistant genotype (CC) was in higher proportion compared to the V1016I resistant genotype (II), which was then followed by the LL genotype at the 410 position (Fig 1). This is in accordance with previous studies that indicate that the V410L mutation has occurred sequentially after the V1016I and 1534C mutations have already been present in a population [27,48]. However, in another study, mosquitoes carrying the V410L mutation were devoid of the F1534C and V1016I genotypes, indicating the V410L mutation can be present independently of the two others [20]. Further, the V410L has been found at higher frequencies compared to the F1534C genes in other countries [49]. In contrast, only in area 53 we found one female of genotype LL/VV/FF and four females VL/VV/FF (Table 2), and in area 73 we found one female of LL/VV/FC. The low frequency of these *kdr* genotypes in Harris County supports a later appearance of the V410L mutation, as was found in the above mentioned studies [27,48].

Of the 23 genotypes found in Harris County for the three *kdr* sites, sixteen carried at least on L allele (Table 2). The L allele appeared most frequently as LL/II/CC genotype the most frequent genotype in each of the areas (48.2%) (Fig 1 and Table 2), followed by the VL/II/CC genotype (19.1%), except for area 75 that only had females of the LL/II/CC genotype. The third most frequent genotype carrying the L mutation was VL/VI/FC (3.8%), although it was not present in areas 75 and 419. The fourth most common genotype was VL/VI/CC (3.4%), not present in areas 45, 75 and 419. The fifth most common genotype with the L allele was LL/VI/FC (2.5%), not present in 23, 75 and 419 (Table 2). It should be noted that only 21 mosquitoes were analyzed from area 419. Area 45 was unusual in that it had 24.4% mosquitoes of the LL/VI/FC genotype, the highest among all areas, which was the second most frequent genotype in that area after LL/II/CC (Table 2). This area supports that the resistant L allele at site 410 could have arisen independently, followed by a crossover event involving established 410L alleles with 1534C alleles, rather than sequential addition of the V1016I mutation to the F1534C, followed by V410L.

### Influence of V410L genotype on survival in field cage tests

In a previous analysis of these field cage tests, we detected differences in survivorship across areas, distances from spray application, and post positions, but not depending on the combined genotypes at the 1016 and 1534 residue sites when comparing them to susceptible mosquitoes [21]. This led us to investigate if the presence of the V410L mutation may contribute to explain the observed differences in survivorship the *Ae*. *aegypti* females. When considering the V410L genotypes only, we found that the homozygous resistant LL had higher survivorship compared to the VL and VV genotypes, which had similar survivorship to each other (S2 Fig). This is similar to reports indicating the V410L mutation segregates as a recessive allele [50]. We also found strong linkage disequilibrium between the V1016I and F1534C genotypes (S3 Table), followed by V410L and V1016I (S1 Table), followed by V410L and F1534C (S2 Table). Within the same operational area linkage disequilibrium was not identical among the three tested *kdr* loci pairs, which may mean that there are different selection forces in the different areas (S1–S3 Tables).

### Influence of distance and genotype on the probability of survival

Females from areas 75, 23, and 419 died in the closest cages at 7.62 m (Fig 1), likely overwhelmed by the amount of pesticide received, as this was the distance where the spray volume was the highest [51]. Conversely, the only females tested at 38.1 m were from area 23 and most survived, as there was likely less pesticide that reached that distance (Fig 1) [51]. This is evidenced by studies in which mosquitoes at further distances survived exposure to pyrethroids regardless of genotype [21, 35].

We first analyzed the independent effect of genotypes at the 410 site on mortality achieved at different distances from the pesticide source (S2 Fig); as well as the effect of the tri-locus *kdr* genotypes on mortality at the different distances (Fig 4). The LL genotype at the 410 site significantly increased survivorship at 15.24- and 22.86-m distances from the application source (S2 Fig). Similarly, a positive effect on survivorship for the homozygous resistant mosquitoes with either the 1016 II genotype or the 1534 CC genotype was observed at the 22.86-m distance.

Compared to the susceptible Orlando Strain females, we observed that the three most frequently occurring genotypes in Harris Co. (VV/II/CC, VL/II/CC, and LL/II/CC) had increased survivorship (Fig 4). This is to be expected as the V1016I and F1534C genotypes are associated with pyrethroid resistance in laboratory assays [24]. However, there were differences in survivorship between the triple resistant (LL/II/CC) and VL/II/CC genotypes at all distances (Fig 4) with the LL/II/CC having higher survival at the two further distances (Fig 4B and 4C). Because we observed differences among the three genotypes by distance, we also wanted to determine if area contributed to these differences.

### Influence of genotype and area on survivorship

Area of origin had a significant negative effect on survivorship for the VL/II/CC genotype only (S4B Fig). These results are mainly driven by area 53, which also had the largest genetic variability, with seventeen of the 23 genotypes (Table 2). We speculate that the lower insecticide selection pressure of only two sprays in the 5 years before this field test, could have decreased other mechanisms of resistance in these mosquitoes (S7 Table). In contrast, area 23 received ten Permanone sprays and area 73, thirteen sprays, and congruent with this higher selection pressure the survivorship of females from both areas was significantly higher than those from area 53 (S7 Table and S4B Fig). For the VL/II/CC genotype the results are similar to a study in California, USA, where area of origin influenced pyrethroid knockdown despite most individual *Ae*. *aegypti* mosquitoes having identical VGSC *kdr* haplotypes [52]. The area of origin should be interpreted as a covariate that includes the different spraying history, and other selective forces that affect the population genetic background beyond the VGSC genotypes, such as other mechanisms of resistance.

In sum, our results support that area, distance, but genotype only at the 15.24- and 22.86-m distances (Fig 4) significantly affect survivorship when including in the analysis the genotype at the V410L site.

### Modeling of factors influencing survivorship

Based on the work of Haddi et al. (2017) [26] that demonstrated the V410L genotype is associated with resistance in *Aedes* mosquitoes in the laboratory, several comprehensive analyses were run to determine factors influencing survivorship, including genotypes at the 410 residue site. When first using a logistic regression analysis that included genotypes at 410 position, distance, and the interaction between those two variables, all three factors influenced survivorship (S4 Table, panel A). We then asked if the genotype at the 410 site in conjunction with the genotypes at 1016 and 1534 contributed to survivorship in the field using a second logistic regression model that included “distance” and the “triple locus genotype” and their interaction (S4 Table, Panel B, top).

Similar to the first model, this second model found that survivorship from the field test was influenced by genotype, distance, and the interaction between distance and tri-locus genotype even when excluding the distance of 38.1 m (area 23 only (S4 Table, panel B, top and middle)). However, when excluding genotypes with low counts, leaving only the VV/II/CC, VL/II/CC, and LL/II/CC genotypes for survival comparisons the effect of genotype alone on survivorship could no longer be detected (S4 Table, B, bottom panel). This indicates the influence of these specific genotypes on survival can only be revealed in the field, as less pesticide is received at increased distances and survivorship is differentially affected by genotype.

Although we detected a total of 23 genotypes in Harris County, the high number of genotypes with low number of mosquito representatives (total females in low count genotypes = 190) made them irrelevant in terms of influencing the overall survivorship with respect to the abundant LL/II/CC genotype. The low count genotypes (L.C.G; ≤ 16 identified) concentrated in areas 53 (15 L.C.G.), 23 (10 L.C.G.), 73 (9 L.C.G.) and 45 (5 L.C.G.) (Table 2); however, these areas still had a preponderance of the LL/II/CC genotype with 35.9%, 35.9%, 38.5% and 46.75% respectively (Fig 1).

The LASSO analysis selects the most significant factors affecting survivorship. From this analysis a forest plot was derived that yielded the odds of survivorship for genotypes by distance in reference to the tri-locus resistant genotype LL/II/CC, and the odds of survivorship depending on area of origin in relationship to the area with the lowest survivorship (area 53) as reference. This plot showed that for the 15.24- and 22.86-m distances, VL/II/CC genotype had decreased survivorship compared to the triple resistant LL/II/CC genotype (Fig 5), indicating that two LL alleles are needed for increased survivorship when the mutation is present at the 410 position. Similar to our observations, in laboratory studies mosquitoes with the heterozygous VL genotype at the 410 site, die at higher rates than the LL genotype but have higher recovery after permethrin knockdown. In contrast, we found that although HCPH personnel maintained the treated mosquitoes from the field tests in the laboratory for 24 h and having scored them 10 min after pesticide application, none of the initially “dead” mosquitoes recovered at 24 h. Not all non-synonymous mutations within the VGSC confer resistance to pyrethroids [53]. When considering the analysis of individual *kdr* genotypes at the 410 site on survivorship in the field, it appears that LL contributed to survivorship, and we did not find differences in mortality between VV and VL (Fig 3).

Intriguingly, there was no difference in survivorship at any distance between the homozygous susceptible (VV/II/CC) and homozygous resistant (LL/II/CC) at the 410 site, likely due to the lower representation of VV/II/CC (n = 72) compared to the LL/II/CC (n = 345) when they were further segregated at each distance. This is evident in Fig 4 where VV/II/CC was statistically intermediate in survivorship at the three distances in comparison to the other genotypes, because their lower number in each distance decreased the statistical power to detect differences.

When considering the analysis of individual *kdr* genotypes at the 410 site on survivorship in the field, it appears that LL contributed to survivorship (Fig 3). However, upon analyzing only the genotypes VV/II/CC, VL/II/CC and LL/II/CC simultaneously, the LL genotype does not appear to increase resistance when present with the II/CC genotype (Fig 5 and S4 Table Panel B, bottom). This is congruent with the results of Fig 4, that at both 15.24- and 22.86-m there are no differences in survivorship between the VV/II/CC and VL/II/CC genotypes and that interactions of these two genotypes with distance are not significant (S6 Table, second and third panel, P value VV/II/CC vs VL/II/CC = 0.055 at 15.24-m and *P* = 0.5746 at 22.86-m). However, the multiple comparisons analyses of the interaction between tri-locus genotypes and distance showed a significant interaction effect, where having an extra L allele (VL/II/CC vs LL/II/CC) at both distances significantly increases the chances of survival (*P* value at 15.24-m < 0.0001: *P* value at 22.86-m < 0.0459) (S6 Table). The LL/II/CC genotype is the only genotype for which the interaction and genotype with distance was significant at all distances, indicating that survivorship of this genotype steadily increased with distance (S6 Table, *P* values for 3 distances each < 0.0001).

Overall, our data support the triple homozygous genotypes appears to have reached a maximum selective advantage at all distances. The results of Fig 3 reflect the fact that the LL genotype is in its majority present in combination with the II/CC genotype.

Other genotype combinations (e.g., VV/VV/FF, LL/VI/FC, etc.) could not be properly analyzed due to low counts of these genotypes, which prevented us from understanding the full effect of each genotype on survival from the field cage tests. Obtaining a congenic strain that only possesses the V410L mutation may provide further insight into the role the V410L genotype plays in resistance. As the mosquitoes used were collected from the field and could not be controlled for genotype, it is likely that there are other resistance mechanisms may be involved.

One mechanism of resistance not analyzed here is metabolic resistance. Overexpression of esterases (ESTs), glutathione s-transferases (GSTs), and cytochrome P450 monooxygenases (P450s), can contribute to pyrethroid resistance [54–56]. The forest plot shows that mosquitoes differed in survivorship based on area of origin (Fig 5). In particular, area 53 had lower survivorship compared to areas 45, 73, 75, and 419 [21]. Notably, area 53 had a significant proportion of dead VL/II/CC mosquitoes compared to other areas (S4 Fig). As the VL mutation is only weakly resistant to permethrin, and we did not find significant differences in survivorship between VV/II/CC and VL/II/CC genotypes at all distances (S4 Table), it may be that females from area 53 lack other resistance mechanisms. The presence of multiple *kdr* mutations within the VGSC may not be sufficient to determine the level of resistance in populations of *Ae*. *aegypti*.

The field cage tests used pyrethroid-susceptible, Orlando-strain females which have been in colony at HCPH-MVCD since 2015, and F_0_ females from different operational areas collected as eggs from the field [21]. While cited limitations of using F_0_ field-collected females as the treatment group include decreased survivorship due altered rearing conditions in the laboratory [57], in our study all the field-collected control females placed upwind (non-treated), survived after 24-h, the end point of the bioassay, indicating they were fit for the test [21]. Furthermore, females collected from the field as eggs consistently survived Permanone 31–66 exposure at higher frequencies compared to the Orlando mosquitoes. These results supported that the females collected from the field as eggs were viable for use in the field cage tests to assess resistance in Harris County. F_0_ females more accurately reflect the genetic diversity present in the field, while the resistant genetic background may be lost during laboratory rearing due to genetic bottlenecks and aggressive breeding selection which can reduce pyrethroid resistance in laboratory colonies in as few as eight generations [58, 59]. However, one challenge of this approach is to obtain a similar number of eggs from all areas. In our study we experienced low representation of females from operational area 23 due to lesser egg collection in the same (Table 2; *N* = 21). In the laboratory another challenge of F_0_ females from field collected eggs required us to synchronize of Orlando-strain female emergence to that of the field collected females [60].

### Conclusions

Most female *Ae*. *aegypti* from Harris County were triple resistant for the V410L, V1016I, and F1534C genotypes despite not being targeted for pyrethroid applications by HCPH-MVCD. The high frequency of the LL/II/CC genotype and high survivorship from the field cage tests after exposure to Permanone 31–66 may pose a public health risk in Harris County. As Harris County applies Permanone 31–66 at the highest allowable rate, these results further indicate that "distance" is crucial for "genotype" to influence survivorship in the field with the LL/II/CC genotype having significant positive interactions at all distances. Insecticide concentrations in the field are known to vary depending on application technique, distance from spray, and environmental conditions [61]. Indeed, insecticide applications remain inadequate beyond 7.62-m distances, even here at the highest label rate authorized. A previous study using congenic LL/II/CC females with a susceptible Rockefeller background had similar levels of survivorship to permethrin as F1534C females, but also increased survivorship to deltamethrin [29]. However, these congenic females have not been field-tested as to prove the influence of this genotype under realistic control scenario. The V410L *kdr* mutation in the *Ae*. *aegypti* population of Harris County adds a risk factor to the control of biting females, as LL/II/CC females are associated with increased resistance to deltamethrin, a type-II pyrethroid [29], however are more susceptible to lambda cyhalothrin [54]. We observed that when in combination with the II/CC genotype, females with the heterozygous VL genotype had lower survivorship compared to LL females. This may mean that possession of the VL genotype without other resistance mechanisms may produce a fitness disadvantage for survival in the field tests. Insecticide resistance can remain as long as ten years in *Aedes* populations without public applications of pyrethroids [62], further complicating control strategies. The high frequencies of the triple resistant genotypes we observed would not be expected unless domestic pesticides are applied by homeowners and private control companies that would be unaccounted for in this study, which should be considered to avoid the increase of resistance in these populations. Currently, there are no alternatives to pyrethroids that can be used at a wide scale and are approved for public use, aside from malathion which is used in Harris County’s rotational strategy for insecticide resistance management. However, malathion is not approved to be applied with a handheld sprayer and concern for cross-resistance remains high.

We have amongst the highest proportion of LL and VL mutations detected and have further supported that possession of resistant genotypes at the 410 site influence of survival from the field cage tests. The selection pressure of pyrethroids in Harris County remains relevant, not only to *Aedes* but *Culex* species in Harris County as well [35]. This resistance poses a threat to chemical-based control strategies in Harris County, highlighting the necessity of novel control strategies for arboviral disease management.

## Supporting information

S1 Fig Percentage of genotypes at the V410L site detected per area across all areas. Across all areas significant differences (Chi-square; *P* < 0.0001) were detected among the proportion of 410 site genotypes when analyzed independently of genotypes at the 1016 and 1534 sites. Horizontal lines above bars indicate areas in which the percentage of genotypes are not significantly different from pairwise comparisons (Fisher Exact test; *P* < 0.05). Black numbers above the bars represent the VV genotype, the grey numbers above the bars represent the VL genotype, and the white numbers in the black bars represent the LL genotype. Area 45 is intermediate between the two groups.
(TIF)

S2 Fig Influence of the *kdr* genotype at the 410 site on survivorship of females at each distance after Permanone 31–66 tests. Panels show results of each of the tested distances from the Permanone 31–66 application source, as follows: (A) 7.62 m, (B) 15.24 m, (C) 22.86 m, and (D) 38.1 m. Asterisks above panels indicate there is significantly different survivorship (Fisher’s Exact Test*; P* < 0.05) among genotypes at that distance. Different letters (a-b) above bars indicate differences in the proportions of surviving females using paired comparisons. White numbers in black bars represent the number of dead females and black numbers above the bars represent the number of live females.
(TIF)

S3 Fig Female survival by tri-locus genotype (410, 1016 and 1534) and post position after Permanone 31–66 tests. Post position refers to the placement of the posts within each distance, where post I is the closest to the beginning of the spraying and post III is the one closest to the ending of the spraying. (A) Post position I, (B) Post position II, and (C) Post position III. Numbers above each bar represent the genotyped mosquitoes that survived, and numbers in white within the black zones are the genotyped mosquitoes that died. Different letters (a-b) above bars indicate differences in the proportions of surviving females (Fisher’s Exact Test*; P* < 0.05).
(TIF)

S4 Fig Influence of operational area of origin on survivorship of the three most frequent genotypes after Permanone 31–66 tests. Panels show survival status of the observed genotypes, as follows: (A) LL/II/CC, (B) VL/II/CC, and (C) VV/II/CC. Numbers above each bar represent the genotyped mosquitoes that survived, and numbers in white within the black zones are the genotyped mosquitoes that died. Different letters (a-b) above bars indicate differences in the proportions of surviving females (Fisher’s Exact Test*; P* < 0.05).
(TIF)

S1 Table Linkage disequilibrium between V410L and V1016I *kdr* genotypes in different operational areas of Harris County. (DOCX)

S2 Table Linkage disequilibrium between V410L and F1534C *kdr* genotypes in different operational areas of Harris County. (DOCX)

S3 Table Linkage disequilibrium between V1016 and F1534C *kdr* genotypes in different operational areas of Harris County. (DOCX)

S4 Table Logistic regression analysis of the genotype at the 410 site (Panel A) and Tri-locus genotypes (Panel B) on survivorship. (DOCX)

S5 Table Multiple comparisons for the interaction between tri-locus genotype and distance using reference level. Information provided here was used to develop Fig 5. (*) Asterisk indicates reference level used for multiple comparisons. Odds ratios are considered significant when the confidence interval of the lower and upper bounds does not contain the value of 1. Number of females used was 526 (see S3 Fig Panel B, bottom).
(DOCX)

S6 Table Multiple comparisons analysis for the interaction between tri-locus genotype and distance. **Bold** indicates *P* value is less than or equal to 0.05.
(DOCX)

S7 Table Number of permethrin sprays performed by Harris County Public Health—Mosquito and Vector Control in areas used for field cage tests (FCT) by time before test date. (DOCX)
