# Supplementary figures and images for: Impact of the V410L kdr mutation and co-occurring genotypes at kdr sites 1016 and 1534 in the VGSC on the probability of survival of the mosquito Aedes aegypti (L.) to Permanone in Harris County, TX, USA
Source: PLoS Negl Trop Dis. 2023 Jan 23;17(1):e0011033. doi: 10.1371/journal.pntd.0011033 (PMC9870149; doi:10.1371/journal.pntd.0011033)

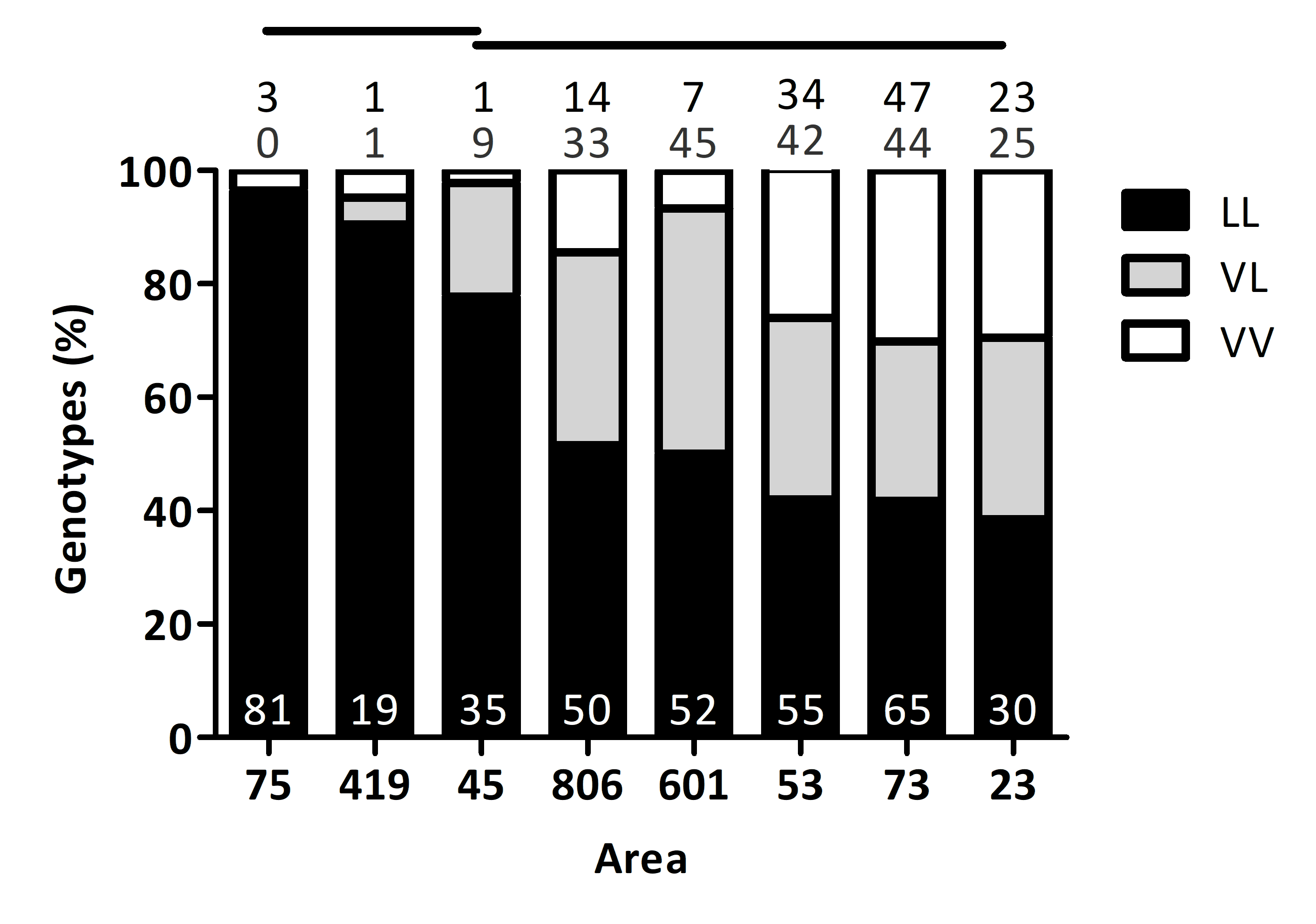

Supplement: S1 Fig — Across all areas significant differences (Chi-square; P < 0.0001) were detected among the proportion of 410 site genotypes when analyzed independently of genotypes at the 1016 and 1534 sites. Horizontal lines above bars indicate areas in which the percentage of genotypes are not significantly different from pairwise comparisons (Fisher Exact test; P < 0.05). Black numbers above the bars represent the VV genotype, the grey numbers above the bars represent the VL genotype, and the white numbers in the black bars represent the LL genotype. Area 45 is intermediate between the two groups. (TIF) [file pntd.0011033.s001.tif]

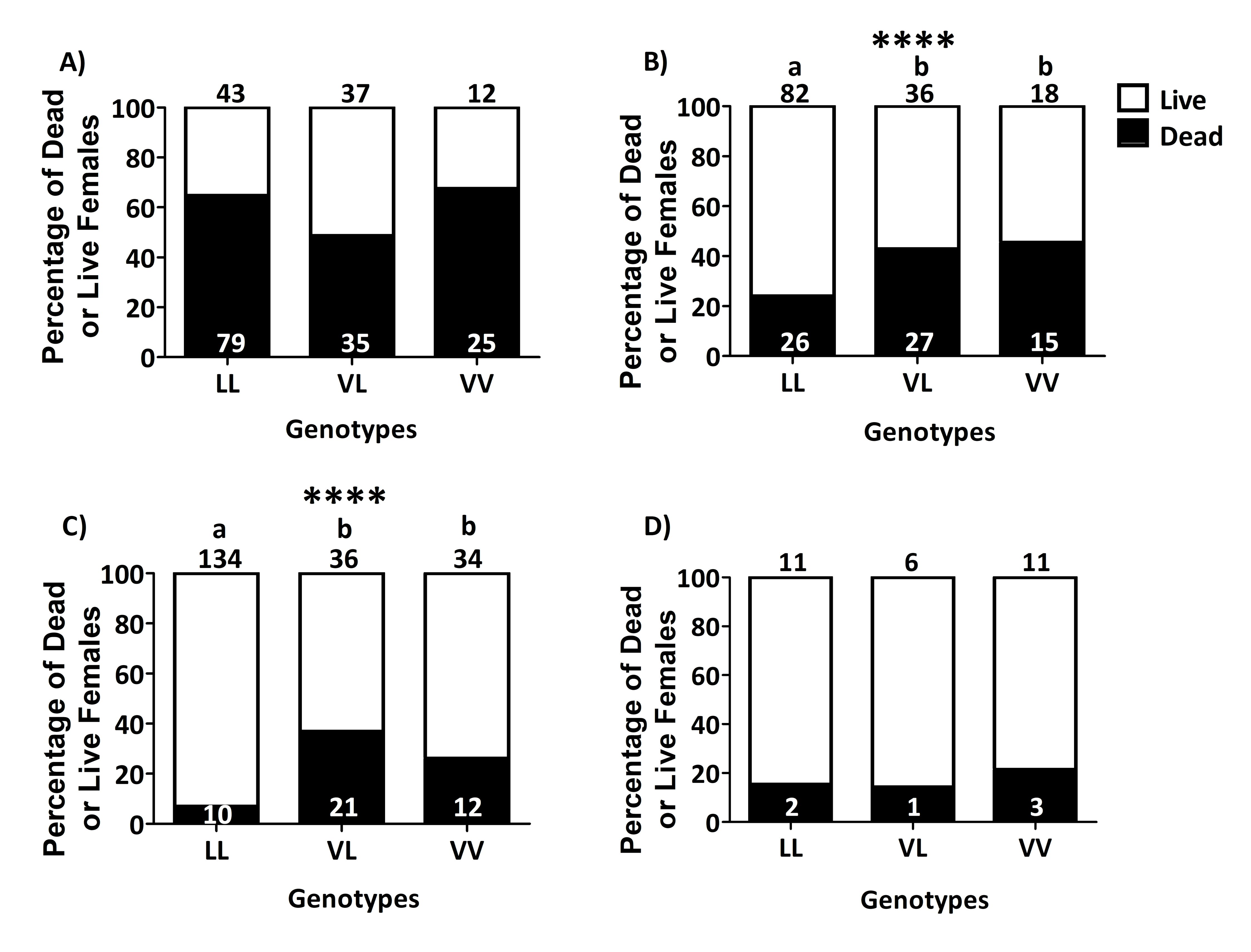

Supplement: S2 Fig — Panels show results of each of the tested distances from the Permanone 31–66 application source, as follows: (A) 7.62 m, (B) 15.24 m, (C) 22.86 m, and (D) 38.1 m. Asterisks above panels indicate there is significantly different survivorship (Fisher’s Exact Test; P < 0.05) among genotypes at that distance. Different letters (a-b) above bars indicate differences in the proportions of surviving females using paired comparisons. White numbers in black bars represent the number of dead females and black numbers above the bars represent the number of live females. (TIF) [file pntd.0011033.s002.tif]

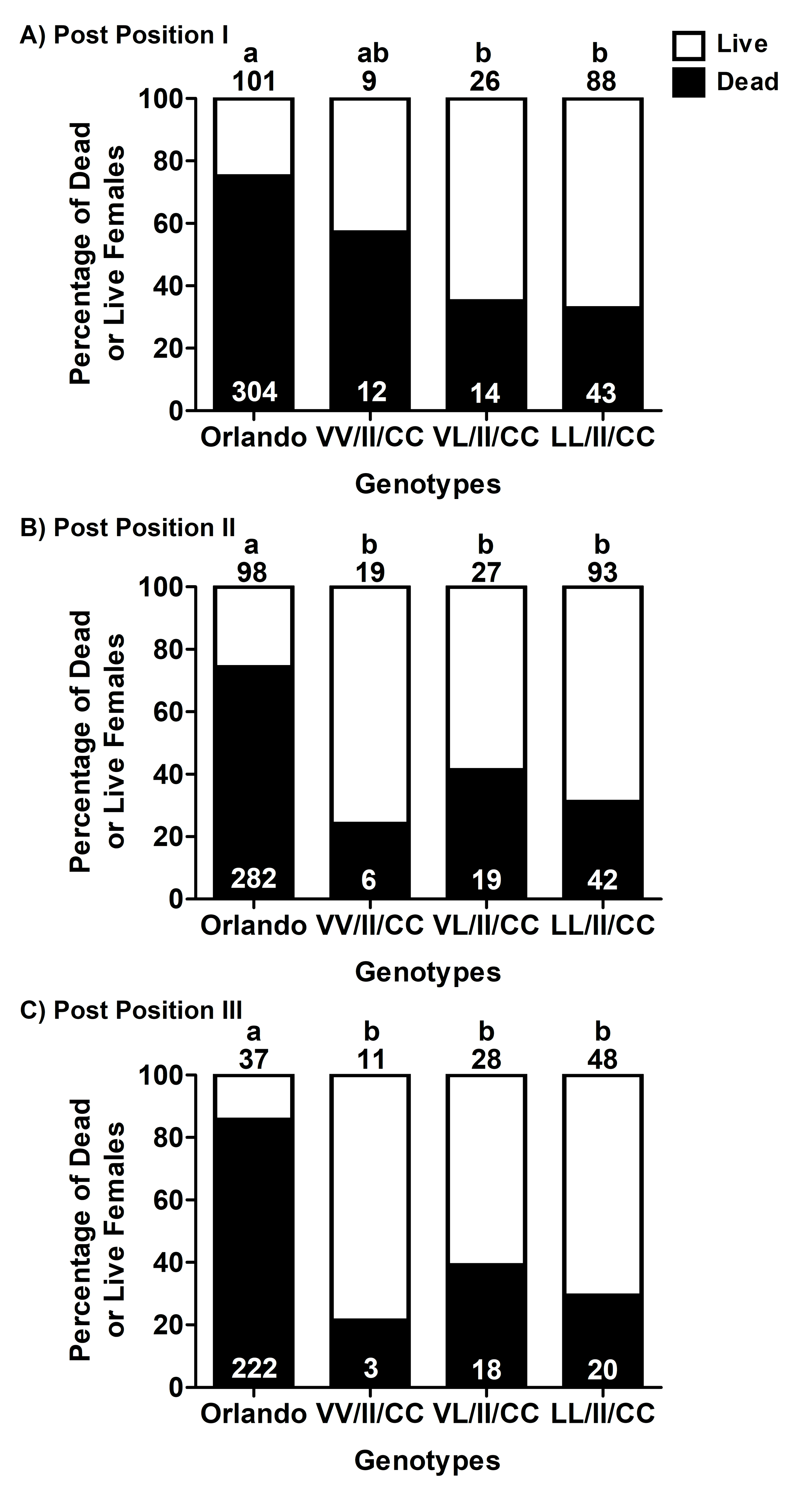

Supplement: S3 Fig — Post position refers to the placement of the posts within each distance, where post I is the closest to the beginning of the spraying and post III is the one closest to the ending of the spraying. (A) Post position I, (B) Post position II, and (C) Post position III. Numbers above each bar represent the genotyped mosquitoes that survived, and numbers in white within the black zones are the genotyped mosquitoes that died. Different letters (a-b) above bars indicate differences in the proportions of surviving females (Fisher’s Exact Test; P < 0.05). (TIF) [file pntd.0011033.s003.tif]

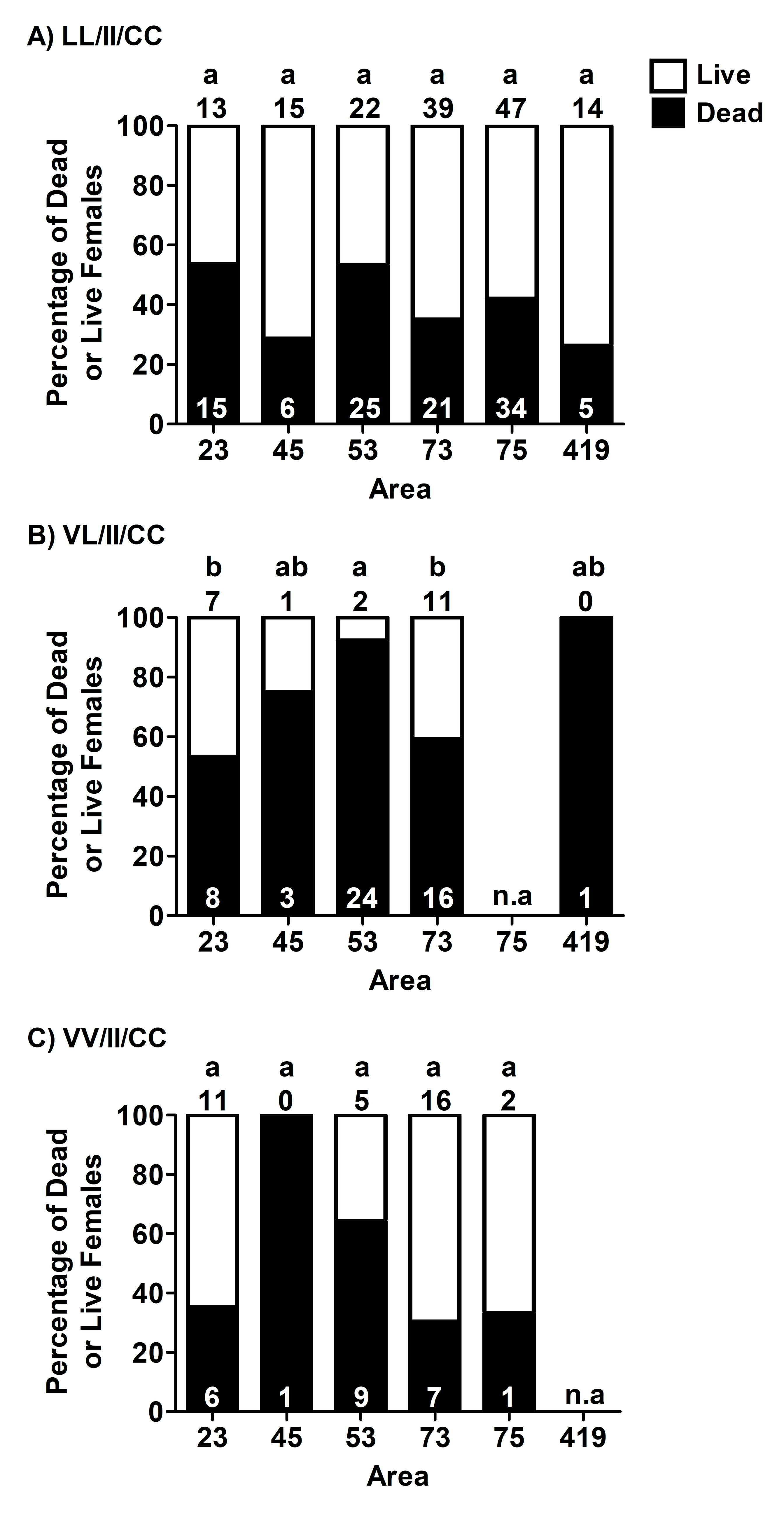

Supplement: S4 Fig — Panels show survival status of the observed genotypes, as follows: (A) LL/II/CC, (B) VL/II/CC, and (C) VV/II/CC. Numbers above each bar represent the genotyped mosquitoes that survived, and numbers in white within the black zones are the genotyped mosquitoes that died. Different letters (a-b) above bars indicate differences in the proportions of surviving females (Fisher’s Exact Test; P < 0.05). (TIF) [file pntd.0011033.s004.tif]
