# Supplementary material for: Impact of the V410L kdr mutation and co-occurring genotypes at kdr sites 1016 and 1534 in the VGSC on the probability of survival of the mosquito Aedes aegypti (L.) to Permanone in Harris County, TX, USA
Source: PLoS Negl Trop Dis. 2023 Jan 23;17(1):e0011033. doi: 10.1371/journal.pntd.0011033 (PMC9870149; doi:10.1371/journal.pntd.0011033)
Supplement: S1 Table — (DOCX) [file pntd.0011033.s005.docx]

**S1 Table. Linkage disequilibrium between V410L and V1016I *kdr* genotypes in different operational areas of Harris County.**

| **Operational Area** | **Date** | **D’** | **Corr.** | **X^2^** | **p-value** | ***N*** |
| --- | --- | --- | --- | --- | --- | --- |
| 23 | 11/9/2017 | 0.872 | 0.356 | 19.716 | < 0.001 | 78 |
| 419 | 9/6/2018 | 0.999 | 0.805 | 27.237 | < 0.001 | 21 |
| 53 | 10/11/2018 | 0.508 | 0.354 | 32.750 | < 0.001 | 131 |
| 73 | 11/6/2018 | 0.710 | 0.409 | 52.140 | < 0.001 | 156 |
| 45 | 7/16/2019 | 0.102 | -0.021 | 0.040 | 0.842 | 45 |
| 75 |  | N/A | N/A | N/A | N/A | 84 |
| 601 | 8/6/2019 | 0.422 | 0.276 | 15.795 | < 0.001 | 104 |
| 806 |  | 0.996 | -0.165 | 5.305 | 0.021 | 97 |
| All Areas |  | 0.537 | 0.330 | 155.901 | < 0.001 | 716 |

*P* values ≤ 0.05 indicate significant disequilibrium.
