# Supplementary material for: Impact of the V410L kdr mutation and co-occurring genotypes at kdr sites 1016 and 1534 in the VGSC on the probability of survival of the mosquito Aedes aegypti (L.) to Permanone in Harris County, TX, USA
Source: PLoS Negl Trop Dis. 2023 Jan 23;17(1):e0011033. doi: 10.1371/journal.pntd.0011033 (PMC9870149; doi:10.1371/journal.pntd.0011033)
Supplement: S2 Table — (DOCX) [file pntd.0011033.s006.docx]

**S2 Table. Linkage disequilibrium between V410L and F1534C *kdr* genotypes in different operational areas of Harris County.**

| **Operational Area** | **Date** | **D’** | **Corr.** | **X^2^** | **p-value** | ***N*** |
| --- | --- | --- | --- | --- | --- | --- |
| 23 | 11/9/2017 | 0.223 | 0.085 | 1.135 | 0.287 | 78 |
| 419 | 9/6/2018 | 0.999 | 0.805 | 27.237 | < 0.001 | 21 |
| 53 | 10/11/2018 | 0.493 | 0.224 | 13.113 | < 0.001 | 131 |
| 73 | 11/6/2018 | 0.828 | 0.286 | 25.554 | < 0.001 | 156 |
| 45 | 7/16/2019 | 0.185 | 0.149 | 1.991 | 0.158 | 45 |
| 75 |  | N/A | N/A | N/A | N/A | 84 |
| 601 | 8/6/2019 | 0.433 | 0.096 | 1.935 | 0.164 | 104 |
| 806 |  | 0.060 | -0.007 | 0.009 | 0.926 | 97 |
| All Areas |  | 0.493 | 0.202 | 58.449 | < 0.001 | 716 |

*P* values ≤ 0.05 indicate significant disequilibrium.
