# Supplementary material for: Impact of the V410L kdr mutation and co-occurring genotypes at kdr sites 1016 and 1534 in the VGSC on the probability of survival of the mosquito Aedes aegypti (L.) to Permanone in Harris County, TX, USA
Source: PLoS Negl Trop Dis. 2023 Jan 23;17(1):e0011033. doi: 10.1371/journal.pntd.0011033 (PMC9870149; doi:10.1371/journal.pntd.0011033)
Supplement: S3 Table — (DOCX) [file pntd.0011033.s007.docx]

**S3 Table. Linkage disequilibrium between V1016 and F1534C *kdr* genotypes in different operational areas of Harris County.**

|  | | | | | | | |  | |
| --- | --- | --- | --- | --- | --- | --- | --- | --- | --- |
| **Operational Area** | **Date** | **D’** | **Corr.** | **X^2^** | **p-value** | ***N*** |  | |  |
| 23 | 11/9/17 | 0.507 | 0.476 | 35.307 | < 0.001 | 78 |  | |  |
| 419 | 9/6/18 | 0.999 | 0.999 | 41.906 | < 0.001 | 21 |  | |  |
| 53 | 10/11/18 | 0.999 | 0.652 | 111.339 | < 0.001 | 131 |  | |  |
| 73 | 11/6/18 | 0.999 | 0.599 | 112.117 | < 0.001 | 156 |  | |  |
| 45 | 7/16/2019 | 0.999 | 0.842 | 63.863 | < 0.001 | 45 |  | |  |
| 75 |  | N/A | N/A | N/A | N/A | 84 |  | |  |
| 601 | 8/6/2019 | 0.997 | 0.340 | 24.034 | < 0.001 | 104 |  | |  |
| 806 |  | 0.557 | 0.369 | 26.475 | < 0.001 | 97 |  | |  |
| All Areas |  | 0.892 | 0.596 | 507.266 | < 0.001 | 716 |  | |  |

*P* values ≤ 0.05 indicate significant disequilibrium.
