# Supplementary material for: Impact of the V410L kdr mutation and co-occurring genotypes at kdr sites 1016 and 1534 in the VGSC on the probability of survival of the mosquito Aedes aegypti (L.) to Permanone in Harris County, TX, USA
Source: PLoS Negl Trop Dis. 2023 Jan 23;17(1):e0011033. doi: 10.1371/journal.pntd.0011033 (PMC9870149; doi:10.1371/journal.pntd.0011033)
Supplement: S4 Table — (DOCX) [file pntd.0011033.s008.docx]

**S4 Table. Logistic regression analysis of the genotype at the 410 site (Panel A) and Tri-locus genotypes (Panel B) on survivorship.**

|  | **A) 410 Site Genotype** | | | | **B) Tri-Locus Genotype** | | | |
| --- | --- | --- | --- | --- | --- | --- | --- | --- |
| **Genotype and Distance** | Variables  (N = 716) | LR Chisq | DF | *P* Value | Variables  (N = 716) | LR Chisq | DF | *P* Value |
|  | V410L | 12.11 | 2 | 0.0023 | Tri-locus | 45.952 | 22 | 0.0020 |
|  | Distance | 104.216 | 3 | <0.0001 | Distance | 105.12 | 3 | <0.0001 |
|  | V410L:Distance | 30.879 | 6 | <0.0001 | Tri-locus:Distance | 56.383 | 30 | 0.0025 |
| **Genotype and Distance by Excluding Distance of 31.8 m** | Variables  (N = 682) | LR Chisq | DF | *P* Value | Variables  (N = 682) | LR Chisq | DF | *P* Value |
|  | V410L | 12.079 | 2 | 0.0024 | Tri-locus | 44.977 | 19 | 0.0007 |
|  | Distance | 97.171 | 2 | <0.0001 | Distance | 102.288 | 2 | <0.0001 |
|  | V410L:Distance | 30.674 | 4 | <0.0001 | Tri-locus:Distance | 54.602 | 26 | 0.0009 |
| **Genotype and Distance by Excluding those with Count ≤ 16** | Variables  (N = 682) | LR Chisq | DF | *P* Value | Variables  (N = 526) | LR Chisq | DF | *P* Value |
|  | V410L | 12.079 | 2 | 0.0024 | Tri-locus | 1.716 | 2 | 0.4240 |
|  | Distance | 97.171 | 2 | <0.0001 | Distance | 93.315 | 2 | <0.0001 |
|  | V410L:Distance | 30.674 | 4 | <0.0001 | Tri-locus:Distance | 22.436 | 4 | 0.0002 |
