# Supplementary material for: Impact of the V410L kdr mutation and co-occurring genotypes at kdr sites 1016 and 1534 in the VGSC on the probability of survival of the mosquito Aedes aegypti (L.) to Permanone in Harris County, TX, USA
Source: PLoS Negl Trop Dis. 2023 Jan 23;17(1):e0011033. doi: 10.1371/journal.pntd.0011033 (PMC9870149; doi:10.1371/journal.pntd.0011033)
Supplement: S5 Table — Information provided here was used to develop Fig 5. (*) Asterisk indicates reference level used for multiple comparisons. Odds ratios are considered significant when the confidence interval of the lower and upper bounds does not contain the value of 1. Number of females used was 526 (see S3 Fig Panel B, bottom). (DOCX) [file pntd.0011033.s009.docx]

**S5 Table.** **Multiple comparisons for the interaction between tri-locus genotype and distance using reference level.** Information provided here was used to develop Fig 5. (*) Asterisk indicates reference level used for multiple comparisons. Odds ratios are considered significant when the confidence interval of the lower and upper bounds does not contain the value of 1. Number of females used was 526 (see S3 Fig Panel B, bottom).

| **Variables** | **Effects** | **Estimate** | **SE** | ***P* Value** | **Odds Ratio** | **Lower Bound** | **Upper Bound** |
| --- | --- | --- | --- | --- | --- | --- | --- |
| (Intercept) |  | -3.2494 | 0.4756 | <0.0001 | - | - | - |
| Triple Locus | VL/II/CC | 0.6104 | 0.6719 | 0.3636 | 1.84 | 0.46 | 6.64 |
|  | VV/II/CC | 0.9733 | 0.7038 | 0.1667 | 2.65 | 0.61 | 10.19 |
|  | LL/II/CC * | - | - | - | 1.00 | - | - |
| Distance (m) | 15.24 | 2.7454 | 0.4383 | <0.0001 | 15.57 | 6.81 | 38.21 |
|  | 22.86 | 4.4277 | 0.5109 | <0.0001 | 83.74 | 32.51 | 242.85 |
|  | 7.62 * | - | - | - | 1.00 | - | - |
| Area | 23 | -0.4102 | 0.6075 | 0.4995 | 0.66 | 0.20 | 2.16 |
|  | 45 | 2.0515 | 0.636 | 0.0013 | 7.78 | 2.28 | 27.83 |
|  | 73 | 1.7135 | 0.4031 | <0.0001 | 5.55 | 2.57 | 12.53 |
|  | 75 | 1.6783 | 0.4673 | 0.0003 | 5.36 | 2.19 | 13.81 |
|  | 419 | 1.9386 | 0.7887 | 0.0140 | 6.95 | 1.57 | 34.27 |
|  | 601 | 21.8509 | 1001.7701 | 0.9826 | >999 | >999 | >999 |
|  | 806 | 7.0207 | 1.1365 | <0.0001 | >999 | 180.79 | >999 |
|  | 53 * | - | - | - | 1.00 | - | - |
| Triple Locus  *Distance | VL/II/CC and 15.24 | -3.9298 | 1.0749 | 0.0003 | 0.02 | 0.00 | 0.15 |
|  | VV/II/CC and 15.24 | -1.2069 | 0.9632 | 0.2102 | 0.30 | 0.05 | 2.09 |
|  | VL/II/CC and 22.86 | -2.3824 | 0.8624 | 0.0057 | 0.09 | 0.02 | 0.52 |
|  | VV/II/CC and 22.86 | -0.9729 | 1.1195 | 0.3848 | 0.38 | 0.05 | 4.17 |
|  | LL/II/CC and 7.62 * | - | - | - | 1.00 | - | - |
