# Supplementary material for: Impact of the V410L kdr mutation and co-occurring genotypes at kdr sites 1016 and 1534 in the VGSC on the probability of survival of the mosquito Aedes aegypti (L.) to Permanone in Harris County, TX, USA
Source: PLoS Negl Trop Dis. 2023 Jan 23;17(1):e0011033. doi: 10.1371/journal.pntd.0011033 (PMC9870149; doi:10.1371/journal.pntd.0011033)
Supplement: S6 Table — Bold indicates P value is less than or equal to 0.05. (DOCX) [file pntd.0011033.s010.docx]

**S6 Table. Multiple comparisons analysis for the interaction between tri-locus genotype and distance. Bold** indicates P-value is less than or equal to 0.05.

| **Tri-Locus** | **Distance** | **Estimate** | **SE** | **P-Value** |
| --- | --- | --- | --- | --- |
| VL/II/CC vs LL/II/CC | 7.62 | 0.6104 | 0.6719 | 0.9915 |
| VV/II/CC vs LL/II/CC | 7.62 | 0.9733 | 0.7038 | 0.8943 |
| VV/II/CC vs VL/II/CC | 7.62 | 0.3629 | 0.8285 | 1.0000 |
| VL/II/CC vs LL/II/CC | 15.24 | -3.3194 | 0.8585 | **<0.0001** |
| VV/II/CC vs LL/II/CC | 15.24 | -0.2336 | 0.6744 | 1.0000 |
| VV/II/CC vs VL/II/CC | 15.24 | 3.0858 | 1.0160 | 0.0550 |
| VL/II/CC vs LL/II/CC | 22.86 | -1.7720 | 0.5732 | **0.0459** |
| VV/II/CC vs LL/II/CC | 22.86 | 0.0004 | 0.8918 | 1.0000 |
| VV/II/CC vs VL/II/CC | 22.86 | 1.7724 | 0.9228 | 0.5746 |
| LL/II/CC | 15.24 vs 7.62 | 2.7454 | 0.4383 | **<0.0001** |
| LL/II/CC | 22.86 vs 7.62 | 4.4277 | 0.5109 | **<0.0001** |
| LL/II/CC | 22.86 vs 15.24 | 1.6823 | 0.4720 | **<0.0001** |
| VL/II/CC | 15.24 vs 7.62 | -1.1844 | 0.9764 | 0.9482 |
| VL/II/CC | 22.86 vs 7.62 | 2.0453 | 0.7394 | 0.1144 |
| VL/II/CC | 22.86 vs 15.24 | 3.2296 | 0.9392 | **0.0151** |
| VV/II/CC | 15.24 vs 7.62 | 1.5385 | 0.8640 | 0.6731 |
| VV/II/CC | 22.86 vs 7.62 | 3.4548 | 1.0292 | **0.0202** |
| VV/II/CC | 22.86 vs 15.24 | 1.9163 | 1.0340 | 0.6227 |
