# Supplementary material for: Impact of the V410L kdr mutation and co-occurring genotypes at kdr sites 1016 and 1534 in the VGSC on the probability of survival of the mosquito Aedes aegypti (L.) to Permanone in Harris County, TX, USA
Source: PLoS Negl Trop Dis. 2023 Jan 23;17(1):e0011033. doi: 10.1371/journal.pntd.0011033 (PMC9870149; doi:10.1371/journal.pntd.0011033)
Supplement: S7 Table — (DOCX) [file pntd.0011033.s011.docx]

**S7 Table. Number of permethrin sprays performed by Harris County Public Health – Mosquito and Vector Control in areas used for field cage tests (FCT) by time before test date.**

| **Area** | **Within 5 Years** | **Within 3 Years** | **Within 1 Year** |
| --- | --- | --- | --- |
| 23 | 10 | 3 | 1 |
| 419 | 3 | 0 | 0 |
| 53 | 2 | 0 | 0 |
| 73 | 13 | 5 | 0 |
| 45 | 3 | 0 | 0 |
| 75 | 5 | 4 | 3 |
| 601 | 9 | 2 | 0 |
| 806 | 8 | 1 | 0 |
